# Cold-water exposure and psychological outcomes in adults: a systematic review and meta-analysis

**DOI:** 10.3389/fphys.2026.1926423

**Published:** 2026-09-16

**Authors:** Steven Ngandeu Schepanski, Florian Batta, Marleen Schröter, Gonza B. Ngoumou, Anna K. Koch, Georg J. Seifert

**Affiliations:** 1 Charité – Universitätsmedizin Berlin, corporate member of Freie Universität Berlin and Hum-boldt-Universität zu Berlin, Charité Competence Center for Traditional and Integrative Medicine (CCCTIM), Berlin, Germany; 2 Charité – Universitätsmedizin Berlin, corporate member of Freie Universität Berlin and Hum-boldt-Universität zu Berlin, Department of Pediatrics, Division of Oncology and Hematology, Berlin, Germany

**Keywords:** affective disorders, cold-water exposure, cold-water immersion, depressive symptoms, mental health, winter swimming

## Abstract

**Introduction:**

Cold-water exposure (CWE), including cold showers, cold-water immersion, and winter swimming, is increasingly discussed as a behavioral strategy for improving mood, stress regulation, and mental wellbeing, but clinical evidence remains fragmented.

**Methods:**

MEDLINE, Embase, PsycINFO, and Web of Science were searched from inception to June 6, 2025, with an update in December 2025. Eligible studies included adults exposed to CWE through cold showers, cold-water immersion, or winter swimming. Randomized, non-randomized controlled, cohort, case-control, and single-group pre-post studies were eligible. Two reviewers independently screened studies, extracted data, and assessed risk of bias. Random-effects multilevel meta-analyses used standardized mean differences, expressed as Hedges g. Sensitivity analyses were conducted where possible.

**Results:**

Thirteen studies including 4105 participants met inclusion criteria. Pooled estimates generally favored CWE for depressive symptoms, perceived stress, wellbeing, and mood or affect, but confidence intervals were wide and several estimates attenuated in controlled-only analyses. Anxiety showed little evidence of consistent benefit. Additional outcomes, including sleep, cortisol, heart rate, and pain, were sparse but generally directionally favorable. Most studies had high, serious, or critical risk of bias.

**Conclusion:**

The available evidence does not establish that CWE improves mood, depressive symptoms, perceived stress, wellbeing, or related outcomes. The evidence remains limited, heterogeneous, and at substantial risk of bias, and several estimates became smaller in controlled only analyses. CWE should not currently be regarded as a standalone treatment for psychiatric disorders. Larger controlled trials are needed to clarify clinical relevance, safety, mechanisms, and optimal use.

**Systematic review registration:**

https://www.crd.york.ac.uk/PROSPERO/, identifier CRD420250654531.

## 1 Introduction

Depression, anxiety, and stress-related symptoms are among the leading causes of disability worldwide and are associated with substantial individual, social, and economic burden (James et al., 2018; Santomauro et al., 2021). Although evidence-based psychotherapies and pharmacological treatments are effective for many individuals, important gaps remain. A considerable proportion of patients experience incomplete response, relapse, or persistent impairment (Rush et al., 2006; Van Dis et al., 2020). Others face barriers to care such as limited-service availability, long waiting times, stigma, and treatment costs (Kazdin and Blase, 2011; Thornicroft et al., 2017). As a result, there is growing interest in accessible, digital, self-directed behavioral strategies that may complement established treatments, increase patient autonomy and self-management, and support mood, stress regulation, and psychological wellbeing (Dineen-Griffin et al., 2019; Kwon et al., 2024; Löchner et al., 2025).

Cold-water exposure (CWE) has roots in longstanding hydrotherapy traditions, including historical accounts from antiquity and its later integration into nineteenth century European naturopathic medicine (Steward, 2002; Tsoucalas et al., 2015). In contemporary contexts, CWE includes practices such as cold showers, cold-water immersion, ice bathing, recreational winter swimming and is increasingly discussed as a behavioral practice for mental and physical health (Flynn et al., 2023; Østergaard et al., 2024; Schepanski et al., 2025). No standardized clinical protocol currently exists. Previous literature has described water temperatures between approx. 7 and 24 °C and exposure durations ranging from 30 seconds to 2 hours, with both single exposures and repeated sessions reported (Schepanski et al., 2025).

Beyond its long-standing use in sport and recovery settings, acute cold exposure elicits pronounced physiological responses (Cain et al., 2025; Castellani and Young, 2016). Repeated exposure has been hypothesized to induce adaptive changes in stress regulation, autonomic functioning, and inflammatory processes (Kox et al., 2014; Spiljar et al., 2021). Voluntary engagement with a physically aversive stimulus may also influence affective functioning through altered interoceptive processing, distress tolerance, and exposure-related learning (Boswell et al., 2013; Carona and Marques, 2024; Craske et al., 2014).

Despite this mechanistic plausibility, also small experimental studies (Huttunen et al., 2004; Kelly and Bird, 2022) have suggested that participants perceive benefits of CWE for mood, stress tolerance, wellbeing, and resilience. However, much of the existing literature has focused on physiological adaptation, athletic performance, or healthy populations, making it difficult to determine whether the findings translate into clinically relevant psychiatric outcomes (Cain et al., 2025). Even when psychological outcomes are reported, studies often differ substantially in exposure modality, temperature, duration, frequency, and participant population. Some also combine CWE with swimming, breathing exercises, mindfulness, or group-based experiences that may independently influence mood and wellbeing (Blades et al., 2024; Ketelhut et al., 2023).

A recent systematic review and meta-analysis (Cain et al., 2025) concluded that the broader evidence base on cold-water immersion and wellbeing is constrained by few randomized trials, small sample sizes, limited population diversity, and difficulty isolating the specific effects of cold exposure from contextual influences such as exercise, social interaction, and natural environment. Unfortunately, this review focused primarily on healthy adults and included a broad range of outcomes spanning cognition, immunity, inflammation, energy, sleep, and general wellbeing, rather than specifically evaluating clinically relevant mental health symptoms.

As a result, it remains unclear whether CWE is associated with changes in validated measures of depressive symptoms, anxiety, perceived stress, mood, sleep, and mental wellbeing across different adult populations, including both healthy individuals and those with clinical conditions. A focused synthesis of these outcomes may help clarify whether the proposed benefits of CWE extend beyond transient changes in subjective wellbeing or recovery-related outcomes and are reflected in measures that are relevant to mental health research and clinical psychiatric practice. Therefore, we conducted a systematic review and meta-analysis to evaluate the association between CWE and validated mental health outcomes in adults. For the purposes of this review, psychological outcomes comprised validated measures of depressive symptoms, anxiety symptoms, perceived stress, general mental wellbeing, mood or affect, and sleep quality.

## 2 Methods

### 2.1 Search strategy and selection criteria

This systematic review and meta-analysis followed the Cochrane Handbook for Systematic Reviews and was reported in accordance with the Preferred Reporting Items for Systematic Reviews and Meta-Analyses (PRISMA) statement (Page et al., 2021). The review protocol was prospectively registered in PROSPERO (CRD420250654531) and subsequently published (Schepanski et al., 2025).

We searched MEDLINE via Ovid, Embase via Ovid, APA PsycINFO via EBSCO, and Web of Science via Clarivate from database inception to June 6, 2025. A targeted update search conducted in December 2025 identified one additional eligible study (Epel et al., 2025). The search strategy combined controlled vocabulary and free-text terms relating to CWE modalities and mental health outcomes. Searches were adapted to the indexing and syntax of each database.

Additional searches were conducted in ClinicalTrials.gov and the World Health Organization International Clinical Trials Registry Platform. Backward and forward citation tracking of included studies and relevant reviews was also performed. No date restrictions were applied. The complete MEDLINE search strategy is provided in the **Supplementary Material** (Search terms).

Records were imported into Rayyan for deduplication and screening. Two reviewers (SNS or MS and FB) independently screened titles, abstracts, and full texts, with disagreements resolved through discussion and adjudication by a third reviewer (AKK) when required. Reasons for exclusion at full-text screening are reported in the Supplementary Material (**Supplementary Table 1**).

Eligible studies quantitatively examined CWE and reported at least one validated mental health outcome in adults aged 18 years or older. Eligible study designs included randomized controlled trials (RCTs), non-randomized controlled studies, cohort studies, case-control studies, and pre-post interventional studies. Cross-sectional studies, case reports, conference abstracts, reviews, and opinion articles were excluded. Studies enrolling elite or professional athletes were excluded to minimize confounding from sport-recovery contexts.

CWE was defined as the umbrella term for structured exposure to water at or below 25 °C delivered via cold showers, cold-water immersion, or recreational winter swimming. Cold-water immersion referred specifically to bodily immersion and included ice baths rather than treating them as separate exposure category. Eligible interventions involved full-body immersion including the head or chest-level immersion at or above the xiphoid process. Both acute single-session exposures and repeated-session protocols were eligible. Studies were excluded if exposure was limited to partial-body immersion, if sauna or passive heating formed part of the exposure sequence, or if exercise immediately preceded or followed cold exposure in a manner consistent with recovery protocols.

### 2.2 Outcomes

Primary outcomes were validated measures of depressive symptoms, anxiety symptoms, perceived stress, general mental wellbeing, and mood or affect. Secondary outcomes included measures of sleep quality, and stress-related physiological outcomes relevant to mental health interpretation, including heart rate, blood pressure, cortisol, and β-endorphins.

Where different instruments were used to measure the same latent construct, outcomes were grouped into prespecified domains. Outcomes reported in fewer than two independent studies were synthesized narratively. Several prespecified physiological, autonomic, neurocognitive, and immune-related outcomes were too sparse or heterogeneous for quantitative synthesis and are summarized in the Supplementary Material (**Supplementary Table 2**).

### 2.3 Data extraction and risk of bias assessment

Data extraction started on 09/05/2025 and was performed independently by two reviewers (SNS or MS and FB) using a piloted standardized extraction form. Extracted variables included study design, participant characteristics, clinical status, intervention modality and parameters, comparator conditions, outcome measures, follow-up time points, and the numerical data required for effect-size estimation.

When multiple post-intervention assessments were reported, the first post-intervention time point was used in the primary quantitative synthesis and later assessments were summarized narratively. When multiple reports described the same study, the most complete publication was used as the primary source and supplemented with information from secondary reports where necessary. Authors were contacted up to two times with a gap of two weeks in between when outcome data were unclear or incomplete.

Risk of bias was assessed independently by two reviewers (SNS or MS and FB) using design-specific tools. RCTs were evaluated using the Cochrane Risk of Bias tool version 1 (RoB1). Non-randomized intervention studies were assessed using Risk Of Bias In Non-randomized Studies of Interventions (ROBINS-I), and observational exposure studies were assessed using Risk Of Bias In Non-randomized Studies - of Exposure (ROBINS-E). Disagreements were resolved through discussion and adjudication by a third reviewer (AKK) when necessary.

A formal GRADE assessment was not specified in the prospectively registered or published protocol. The review was designed as an exploratory synthesis across randomized, nonrandomized, uncontrolled, and observational evidence rather than around one uniform intervention comparison. We therefore did not retrospectively construct separate GRADE assessments after completion of the analyses. Confidence in the evidence was interpreted qualitatively with explicit consideration of risk of bias, inconsistency, imprecision, indirectness, and dependence on uncontrolled evidence.

### 2.4 Statistical analysis

Meta-analyses were conducted when at least two independent studies reported data for a given outcome domain. Continuous outcomes were summarized as standardized mean differences (Hedges g). In controlled studies, effect sizes represented between-group differences in pre-post change wherever change-score data were available. In single-group studies, effect sizes represented within-group pre-post change. When change-score standard deviations were not reported, they were derived from available summary statistics or reconstructed from pre- and post-intervention values using prespecified assumptions, including an assumed pre-post correlation described in detail in the Supplementary Material (Other Methods). Effect directions were harmonized so that negative values reflected reductions in depression, anxiety, and stress symptoms, whereas positive values reflected improvements in wellbeing, mood, and sleep outcomes.

Random-effects models were used throughout. Because some domains included more than one effect contributed at the study level and because we wished to retain a consistent analytic framework across outcomes, multilevel random-effects meta-analysis was used as the default approach, with study specified as a random factor. Models were estimated using restricted maximum likelihood. Given the small number of studies per domain, inference was based on t-distribution methods with small-sample adjustment.

For each primary outcome domain, we fitted two principal models, namely an overall model including all eligible study designs and a controlled-only sensitivity model excluding single-group pre-post studies. The controlled-only analyses were prespecified as the most important design-based sensitivity analyses because uncontrolled designs were considered more vulnerable to bias and could inflate apparent effects.

Between-study heterogeneity was summarized using τ², I², and Cochran’s Q statistic. Where estimable, prediction intervals were calculated to reflect the range of effects expected in similar future studies. Formal meta-regression, subgroup, and dose-response analyses were not performed because outcome domains generally included only two to five studies, making such analyses underpowered and unstable.

Sensitivity analyses consisted of leave-one-out analyses for the main model and for the controlled-only model. In addition, for the primary outcomes, Bayesian hierarchical sensitivity analyses were conducted to examine the robustness of pooled estimates to alternative assumptions about between-study heterogeneity. These analyses were intended to evaluate the stability of the estimated effect direction and magnitude under plausible prior structures rather than to replace the primary frequentist models.

Formal assessment of publication bias was not undertaken because funnel plots and regression-based methods are unreliable when only a small number of studies are available. Forest plots were used to display pooled estimates, and risk-of-bias assessments were visualized using traffic-light plots. All analyses were conducted in R.

## 3 Results

The study selection process is outlined in **Figure 1**. We screened 18752 records and assessed 19 full-text articles for eligibility (**Table 1**). Thirteen studies met inclusion criteria and were included in the review, comprising five RCTs (Blades et al., 2024; Buijze et al., 2016; Epel et al., 2025; Ketelhut et al., 2023; Slezakova et al., 2025), two non-randomized controlled studies (Hinkka et al., 2017; Petraskova Touskova et al., 2022), four single-group interventional studies (Faid et al., 2022; Knill-Jones et al., 2025; Reed et al., 2023; Yankouskaya et al., 2023), and two observational cohort studies (Hirvonen et al., 2002; Huttunen et al., 2001).

**Figure 1:**
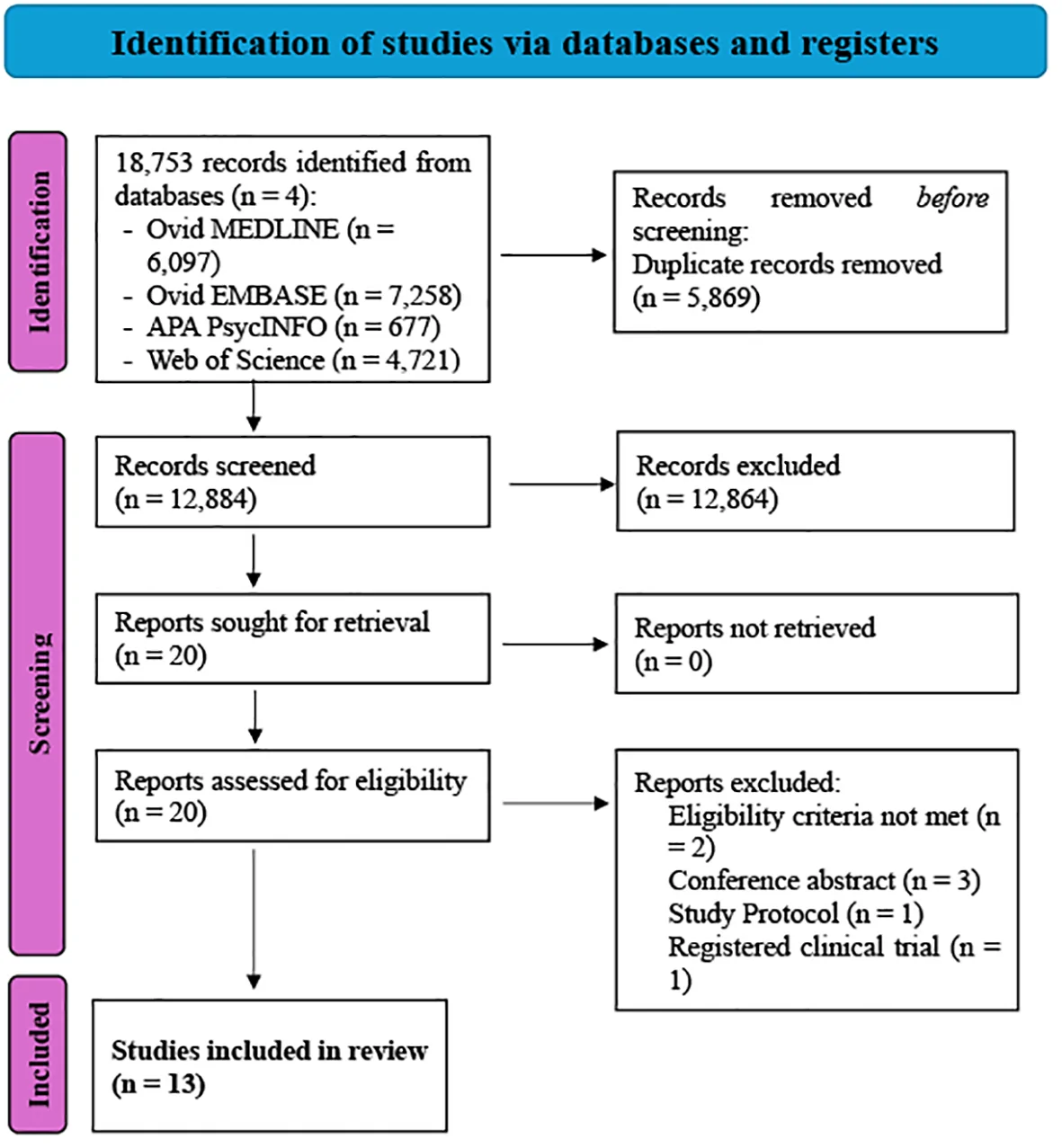
PRISMA flow diagram of study selection.

**Table 1:** Characteristics of Included Studies.

Authors, year	Country	Design	Sample size	Age	Female (%)	Health status	CWE modality	Co-intervention	Control type	Outcome domains	Adherence
(Blades et al., 2024)	United States of America	RCT	43 (IG); 41 (CG)	Mean 43.5	100	Moderate depressive symptoms	Cold shower	Breathing component	Active control	Depression, anxiety, stress, mood, physiological stress markers, adverse events	Monetary incentives; daily questionnaires
(Buijze et al., 2016)	Netherlands	RCT	798 (30s); 727 (60s); 775 (90s); 718 (CG)	Mean 39.4 (IGs), 39.2 (CG)	59 (30s); 58 (60s); 60 (90s); 56 (CG)	Healthy	Cold shower	–	Usual routine	General mental health, wellbeing, sickness absence	Weekly reminders
(Ketelhut et al., 2023)	Switzerland	RCT	22 (IG); 20 (CG)	Mean 26.5 (IG); 26.9 (CG)	0	Healthy	Cold shower	Mind-body-medicine component	No treatment	Stress, affect, cardiovascular outcomes, pain	Daily logs
(Slezakova et al., 2025)	Slovakia	RCT	15 (IG); 15 (CG)	Median 40 (IG), 38 (CG)	54.5 (IG); 69.2 (CG)	Multiple sclerosis	Mixed cold exposure	Multimodal wellness component	No treatment	Depression, anxiety, adverse events	Not reported
(Faid et al., 2022)	United Kingdom	Non-randomized interventional study	48 (IG only)	Not reported	55.2	Healthy	Cold shower	Mind-body-medicine component	–	Stress, depression, wellbeing	Daily reminders; diary template
(Hinkka et al., 2017)	Finland	Non-randomized interventional study	156 (IG); 156 (CG)	Not reported	75.2	Rheumatic diseases	Cold shower	Rehabilitation setting	Active control	Depression, sleep, pain, cardiovascular outcomes	Built into rehabilitation schedule
(Hirvonen et al., 2002)	Finland	Observational cohort	25 (IG); 11 (CG)	Mean 55.1 (IG); 53.8 (CG)	72 (IG); 72.7 (CG)	Healthy	Winter swimming	Naturalistic CWE	No treatment	Mood, alexithymia, cardiovascular outcomes, neuroendocrine markers	Not reported
(Huttunen et al., 2001)	Finland	Observational cohort	49 (IG); 33 (CG)	Median 53 (IG); 51 (CG)	80.6 (IG); 73.9 (CG)	Mixed chronic conditions	Winter swimming	Naturalistic CWE	No treatment	Mood, memory	Not reported
(Knill-Jones et al., 2025)	United Kingdom	Non-randomized interventional study	13 (IG only)	Mean 20.85	38.5	Healthy	Cold water immersion	–	–	Wellbeing, anxiety, sleep, cognition, thermoregulation	Familiarization session
(Petraskova Touskova et al., 2022)	Czech Republic	Non-randomized interventional study	6 (IG); 7 (CG)	Mean 36.4	33.3 (IG); 14.3 (CG)	Healthy	Cold water immersion	Mind-body-medicine component	No treatment	Trauma symptoms, depression, physiological stress markers	Supervised sessions; logs
(Reed et al., 2023)	United States of America	Non-randomized interventional study	16 (IG only)	Mean 23	43.8	Healthy	Cold water immersion	–	–	Affect, cardiovascular outcomes, physiological stress markers, thermoregulation	Supervised laboratory sessions
(Yankouskaya et al., 2023)	United Kingdom	Non-randomized interventional study	33 (IG only)	Mean 28.36	48.5	Healthy	Cold water immersion	–	–	Affect, cardio respiratory responses, neuroimaging outcomes	Physiological monitoring
(Epel et al., 2025)	United States of America	Mixed design	37 (IG); 44 (CG)	Range 30 to 60	100	Healthy	Cold shower	Breathing component	Active control	Depression, stress, mood, physiological stress markers, adverse events	Monetary incentives; daily questionnaires

CWE, cold water exposure; RCT, randomized controlled trial; IG, intervention group; CG, control group. Intervention categories were harmonized to emphasize the core CWE component. “Mind body component” included breathing exercises, meditation, mindfulness, imagery, or related practices. “Multimodal wellness component” included combinations of breathing, mindfulness, imagery, physical movement, or partial immersion procedures.

The included studies examined CWE across a range of adult populations, most commonly healthy volunteers, with a minority recruiting clinical samples such as individuals with depression (Blades et al., 2024), multiple sclerosis (Slezakova et al., 2025), or rheumatic disease (Hinkka et al., 2017) (**Table 1**). Sample sizes ranged from 13 to 3018 participants. The included studies evaluated cold showers, cold water immersion, winter swimming, and mixed cold exposure programs. Among studies reporting the relevant information, temperatures ranged from approximately 6 to 7 °C to 20 °C and session durations ranged from 30 seconds to 15 minutes. Exposure frequency ranged from a single session to twice daily practice, while total intervention periods ranged from a single session to an entire winter season. Interventions were conducted at home, in laboratories, in rehabilitation settings, and outdoors and varied from unsupervised to fully supervised delivery. Several programs combined CWE with breathing exercises, meditation, mindfulness, or swimming. Comparator conditions included warm water exposure, low arousal breathing, usual care, nonexposed controls, and no treatment (**Table 2**).

**Table 2:** Interventions description.

Authors, year	CWE modality	Temperature	Session duration	Frequency	Progression	Control type	Supervision	Setting	Total duration
(Blades et al., 2024)	Cold shower	Not reported; coldest tolerable	Brief to moderate (30s to 3 min)	Daily	Progressive increase	Active control	Unsupervised	Home based	21 days
(Buijze et al., 2016)	Cold shower	≈ 10-12 °C	Brief (30-90s)	Daily	Gradual self-directed increase	Usual routine	Unsupervised	Home based	30 days + follow-up
(Ketelhut et al., 2023)	Cold shower	≈ 14 °C	Brief, increasing over time	Daily	None	No treatment	Unsupervised	Home based	15 days
(Slezakova et al., 2025)	Mixed cold exposure	Mixed, including ≈ 6-7 °C river immersion	Brief to moderate	Multiple times/ week	None	No treatment	Partially supervised	Mixed home and group-based setting	84 days
(Faid et al., 2022)	Cold shower	Not reported	Brief (30-90s)	Daily	None	–	Unsupervised	Home based	10 days
(Hinkka et al., 2017)	Cold shower	Not reported	Short (2 min)	Twice daily	None	Active control	Supervised	Inpatient rehabilitation	2x5 days periods
(Hirvonen et al., 2002)	Winter swimming	Not reported	Not reported	5-6 times / week	None	No treatment	Unsupervised	Outdoor	Entire winter season
(Huttunen et al., 2001)	Winter swimming	Not reported	Not reported	≈ 4 times/ week	None	No treatment	Unsupervised	Outdoor	≈ 4 months
(Knill-Jones et al., 2025)	Cold water immersion	≈ 10.5 °C	Moderate (10 min)	3 times/ week	None	–	Supervised	Laboratory-based	≈ 4 weeks
(Petraskova Touskova et al., 2022)	Cold water immersion	Not reported	Not reported	Daily	None	No treatment	Partially supervised	Outdoor	≈ 8 weeks
(Reed et al., 2023)	Cold water immersion	≈ 10.5 °C	Moderate (15 min)	Single session	None	–	Supervised	Laboratory-based	Single session
(Yankouskaya et al., 2023)	Cold water immersion	≈ 20 °C	Brief (5 min)	Single session	None	–	Supervised	Laboratory-based	Single session
(Epel et al., 2025)	Cold shower	Coldest available temperature	Brief to moderate (30 s to 3 min)	Daily	Progressive increase	Active control	Unsupervised	Home based	21 days

CWE, cold water exposure.

### 3.1 Primary outcomes

Psychological outcomes were available for depressive symptoms, anxiety, perceived stress, wellbeing, and mood or affect, although the evidence base differed across domains in both study design and sample composition (**Figure 2**). Depressive symptoms were the most broadly represented psychological outcome domain, contributing six studies (Blades et al., 2024; Epel et al., 2025; Faid et al., 2022; Hinkka et al., 2017; Petraskova Touskova et al., 2022; Slezakova et al., 2025) and 457 participants, followed by mood or affect with five studies (Epel et al., 2025; Huttunen et al., 2001; Ketelhut et al., 2023; Reed et al., 2023; Yankouskaya et al., 2023) and 492 participants, perceived stress with four studies (Blades et al., 2024; Epel et al., 2025; Faid et al., 2022; Ketelhut et al., 2023) and 218 participants, anxiety with three studies (Blades et al., 2024; Buijze et al., 2016; Knill-Jones et al., 2025; Slezakova et al., 2025) and 115 participants, and wellbeing with two studies (Faid et al., 2022; Knill-Jones et al., 2025) and 42 participants. Additional psychological outcomes such as alexithymia, stress rumination, memory, and quality of life were each assessed in single studies (Blades et al., 2024; Buijze et al., 2016; Epel et al., 2025; Hirvonen et al., 2002; Huttunen et al., 2004; Ketelhut et al., 2023; Knill-Jones et al., 2025; Petraskova Touskova et al., 2022; Reed et al., 2023; Yankouskaya et al., 2023) and were summarized narratively (Supplementary Material, **Supplementary Table 2**).

**Figure 2:**
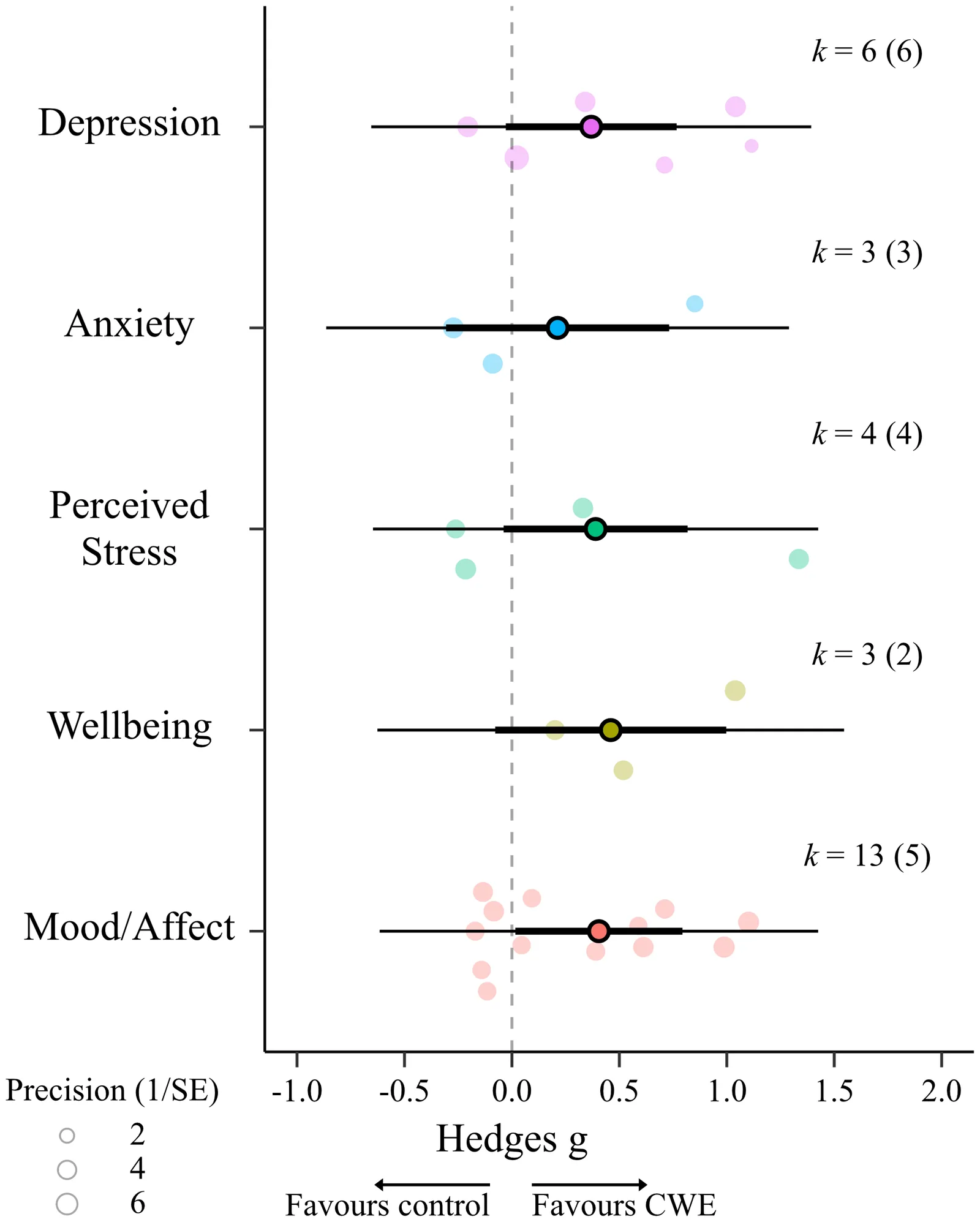
Orchard plot of primary psychological outcomes associated with CWE. Positive Hedges g values indicate effects in the favorable direction for CWE, whereas negative values indicate effects favoring the comparator condition. For visual comparability across domains, symptom-based outcomes including depression, anxiety, and perceived stress were sign-reversed so that positive values consistently reflect lower symptom severity. Colored circles represent pooled estimates for each outcome domain and horizontal lines indicate corresponding 95% confidence intervals. Smaller semi-transparent circles represent individual study effect sizes. Circle size reflects study precision, expressed as the inverse of the standard error (1/SE), with larger circles indicating more precise estimates. Labels indicate the number of effect sizes and contributing studies for each outcome domain, presented as k (studies).

For depressive symptoms, the main multilevel random-effects model including all eligible study designs suggested a small-to-moderate reduction associated with CWE (g = -0.42, 95% CI -0.99 to 0.14) (**Supplementary Figure 1**), although uncertainty was substantial. The controlled-only sensitivity analysis attenuated to a smaller estimate (g = -0.20, 95% CI -0.74 to 0.33) (**Supplementary Figure 2**), indicating that the overall pooled effect was partly influenced by the inclusion of an uncontrolled single-group study. Leave-one-out analyses showed that the pooled estimate remained negative across all models, ranging from -0.55 to -0.20 in the all-design analysis and from -0.35 to -0.10 in the controlled-only analysis (**Supplementary Figures 3**, **4**). Heterogeneity sensitivity analyses likewise yielded consistently negative estimates, ranging from -0.45 to -0.25 (**Supplementary Figure 5**).

For anxiety, the main model showed little evidence of a consistent association between CWE and anxiety symptoms (g = -0.07, 95% CI -1.45 to 1.31) (**Supplementary Figure 6**). The controlled-only analysis remained similarly uncertain (g = -0.23, 95% CI -7.33 to 6.87) (**Supplementary Figure 7**). Sensitivity analyses were less stable than for depression. Leave-one-out analyses yielded both slightly favorable and slightly adverse estimates, and heterogeneity sensitivity analyses ranged from 0.05 under no heterogeneity to -0.11 under higher heterogeneity assumptions (**Supplementary Figures 8**–**10**). Taken together, these findings did not support a robust signal for anxiety.

For perceived stress, the main multilevel model suggested a reduction in stress in favor of CWE (g = -0.30, 95% CI -1.48 to 0.88) (**Supplementary Figure 11**), but the controlled-only analysis shifted essentially to the null (g = 0.03, 95% CI -0.79 to 0.86) (**Supplementary Figure 12**). In leave-one-out analyses, the pooled estimate remained negative in all but the model excluding the uncontrolled study, where the estimate moved slightly above zero (**Supplementary Figures 13**, **14**). Heterogeneity sensitivity analyses, however, consistently remained in the favorable direction, ranging from -0.32 to -0.30 (**Supplementary Figure 15**). The perceived stress estimate was sensitive to study design and did not provide conclusive evidence of an association with CWE.

For wellbeing, the pooled point estimate was positive, but the confidence interval was extremely wide and included the null value (g = 0.68, 95% CI -3.69 to 5.06) (**Supplementary Figure 16**). This domain was represented by only two uncontrolled studies and no controlled only synthesis was possible. Leave-one-out analyses remained positive, with estimates ranging from 0.35 to 1.04, and heterogeneity sensitivity analyses likewise remained positive, ranging from 0.64 to 0.69 (**Supplementary Figures 17**, **18**). The available evidence was insufficient to determine whether CWE was associated with improved wellbeing.

Mood or affect was assessed more heterogeneously than the other psychological outcomes, with several studies contributing multiple subscale-based effect sizes reflecting different affective states or mood dimensions. In the main multilevel model, mood/affect showed a small-to-moderate improvement in favor of CWE (g = 0.36, 95% CI -0.14 to 0.86) (**Supplementary Figure 19**). Restriction to controlled studies attenuated the estimate to g = 0.15 (95% CI -0.08 to 0.39) (**Supplementary Figure 20**). Leave-one-out analyses showed that the pooled estimate remained positive across all models, ranging from 0.17 to 0.44 in the all-design analysis and from 0.10 to 0.19 in the controlled-only analysis (**Supplementary Figures 21**, **22**). Heterogeneity sensitivity analyses also yielded consistently positive estimates, ranging from 0.33 to 0.36 (**Supplementary Figure 23**). The mood or affect point estimates remained positive across the sensitivity analyses, but the evidence remained statistically inconclusive and did not establish efficacy.

### 3.2 Secondary outcomes

Additional outcomes included sleep, cortisol, β-endorphin, systolic blood pressure, diastolic blood pressure, heart rate, and pain, although these domains were generally represented by few studies and small evidence bases. Sleep was assessed in two studies and 255 participants, cortisol in three studies (Blades et al., 2024; Petraskova Touskova et al., 2022; Reed et al., 2023) and 94 participants, β-endorphin in two studies (Hirvonen et al., 2002; Reed et al., 2023) and 38 participants, systolic blood pressure in four studies (Hinkka et al., 2017; Hirvonen et al., 2002; Ketelhut et al., 2023; Reed et al., 2023) and 335 participants, diastolic blood pressure in three studies (Hinkka et al., 2017; Ketelhut et al., 2023; Reed et al., 2023) and 299 participants, heart rate in three studies (Hinkka et al., 2017; Ketelhut et al., 2023; Reed et al., 2023) and 299 participants, and pain in two studies (Hinkka et al., 2017; Ketelhut et al., 2023) and 283 participants.

For sleep, the pooled estimate suggested a small improvement in favor of CWE (g = -0.17, 95% CI -0.30 to -0.05) (**Supplementary Figure 24**). The controlled-only estimate was nearly identical, and both leave-one-out and heterogeneity sensitivity analyses remained consistently negative, suggesting a directionally stable but sparse evidence base (**Supplementary Figures 25**–**27**).

For cortisol, the main model suggested a small reduction in favor of CWE (g = -0.35, 95% CI -0.81 to 0.12), with the controlled-only analysis yielding a similar estimate (g = -0.41, 95% CI -2.22 to 1.40) (**Supplementary Figures 28**, **29**). Leave-one-out and heterogeneity sensitivity analyses likewise remained consistently negative, indicating a directionally favorable but imprecise pattern (**Supplementary Figures 30**–**32**).

For β-endorphin, the pooled estimate was small and slightly unfavorable to CWE (g = 0.13, 95% CI -2.29 to 2.54) (**Supplementary Figure 33**). The controlled-only estimate was somewhat larger in the same direction, although this analysis was based on a single study only (**Supplementary Figure 34**). Sensitivity analyses were less stable than for most other outcomes, with leave-one-out analyses changing direction depending on the study removed (**Supplementary Figures 35**, **36**).

For pain, the pooled estimate suggested a small reduction in favor of CWE (g = -0.24, 95% CI -1.72 to 1.24), showing identical results for controlled-only sensitivity analysis, because both studies were controlled (**Supplementary Figure 37**). Sensitivity analyses also remained consistently negative, although the evidence base was limited (**Supplementary Figures 38**, **39**).

For systolic and diastolic blood pressure, the findings did not support a consistent benefit of CWE. Systolic blood pressure showed a small adverse estimate in the main model (g = 0.21, 95% CI -0.46 to 0.89), which attenuated almost entirely in the controlled-only analysis (g = 0.04, 95% CI -0.13 to 0.20) (**Supplementary Figures 40**, **41**). Diastolic blood pressure likewise showed no clear pattern, with the main model slightly unfavorable to CWE (g = 0.20, 95% CI -1.21 to 1.61) but the controlled-only analysis shifting slightly in the favorable direction (g = -0.10, 95% CI -0.25 to 0.05) (**Supplementary Figures 42**, **43**). Across both blood pressure outcomes, leave-one-out and heterogeneity sensitivity analyses indicated unstable estimates (**Supplementary Figures 44**–**49**). For heart rate, the pooled estimate suggested a moderate reduction in favor of CWE (g = -0.52, 95% CI -1.76 to 0.71) (**Supplementary Figure 50**), with the controlled-only analysis showing a somewhat smaller but similarly favorable estimate (g = -0.32, 95% CI -1.24 to 0.59) (**Supplementary Figure 51**). Leave-one-out and heterogeneity sensitivity analyses remained consistently negative (**Supplementary Figures 52**–**54**), suggesting a relatively stable directional pattern despite the small evidence base.

Overall, the evidence for these additional outcomes was sparse. Point estimates for sleep, cortisol, heart rate, and pain were in the favorable direction, whereas β endorphin and blood pressure outcomes showed no consistent pattern. With the exception of the pooled sleep estimate, the confidence intervals included the null value. These findings should be interpreted descriptively rather than as evidence of efficacy.

### 3.3 Safety and adverse events

Safety and adverse events were reported inconsistently (Buijze et al., 2016). reported that no serious adverse events were considered related to the cold shower intervention, although prolonged sensations of cold in the body or extremities were frequently described. Numerical incidence data were not available in an extractable format. The remaining studies did not provide sufficiently consistent information to estimate the frequency or severity of adverse events across the evidence base.

### 3.4 Risk of bias

Given the small evidence base and the reliance on uncontrolled designs in several domains, interpretation of these findings also requires consideration of study quality and risk of bias. Most studies were judged to have a high, serious, or critical overall risk of bias. Among the five RCTs (Blades et al., 2024; Buijze et al., 2016; Epel et al., 2025; Ketelhut et al., 2023; Slezakova et al., 2025) assessed with the RoB1, all received an overall high-risk judgement (Supplementary Material, **Supplementary Figure 55**). This was driven primarily by high risk in the blinding of participants and personnel domain, which was judged as high risk in all studies, and by additional concerns regarding incomplete outcome data and other potential sources of bias. However, the absence of participant blinding is difficult to avoid in behavioral interventions such as CWE, where participants are inherently aware of whether they are entering cold water. Consequently, these overall judgements may partly reflect the limited fit of conventional risk-of-bias frameworks for non-pharmacological and experiential interventions rather than solely poor methodological quality.

The six non-randomized interventional studies (Faid et al., 2022; Hinkka et al., 2017; Knill-Jones et al., 2025; Petraskova Touskova et al., 2022; Reed et al., 2023; Yankouskaya et al., 2023) assessed with ROBINS-I were judged to have either serious or critical risk of bias (Supplementary Material, **Supplementary Figure 56**). The two observational cohort studies (Hirvonen et al., 2002; Huttunen et al., 2001) assessed with ROBINS-E were both judged to be at very high overall risk of bias, primarily due to concerns about confounding and participant selection (Supplementary Material, **Supplementary Figure 57**). The most common concerns were bias due to confounding, deviations from intended interventions, and outcome measurement.

## 4 Discussion

The pooled point estimates frequently favored CWE, but the current evidence remains too limited and heterogeneous to support conclusions about psychological or clinical benefit. Confidence intervals were often wide, most included the null value, and several estimates became smaller when analyses were restricted to controlled studies. The controlled only findings therefore provide no clear evidence that CWE improves the primary psychological outcomes assessed in this review.

The direction of a pooled point estimate should not be interpreted as evidence of efficacy or clinical relevance. Most confidence intervals included the null value and several estimates became smaller in controlled only analyses. In addition, the use of standardized mean differences across different instruments does not establish whether participants experienced a clinically meaningful improvement. The findings should therefore be regarded as preliminary and hypothesis generating.

For depressive symptoms (Blades et al., 2024; Epel et al., 2025; Faid et al., 2022; Hinkka et al., 2017; Petraskova Touskova et al., 2022; Slezakova et al., 2025), point estimates remained negative across the main model and sensitivity analyses. However, the confidence intervals included the null value and the estimate became smaller when the analysis was restricted to controlled studies. These findings do not establish that CWE reduces depressive symptoms. Mood or affect (Epel et al., 2025; Huttunen et al., 2001; Ketelhut et al., 2023; Reed et al., 2023; Yankouskaya et al., 2023) also showed relatively consistent favorable effects, although these outcomes were more heterogeneous because studies often contributed multiple subscale-based effect sizes covering vigor, fatigue, tension, anger, and positive affect.

The distinction between depressive symptoms and mood related outcomes may help explain some inconsistency in the evidence. Mood, affect, vitality, and wellbeing measures capture broader subjective experiences such as energy, positive functioning, optimism, and perceived flourishing, whereas depressive symptom measures assess more persistent and clinically relevant distress (Lyubomirsky and Lepper, 1999; Tennant et al., 2007). It is plausible that CWE exerts relatively immediate effects on arousal, energy, vitality, and positive mood while producing smaller and less consistent changes in more established psychiatric symptoms. This interpretation is broadly consistent with reports of improvements in mood disturbance, vigor, and esteem related affect following a single cold-water immersion, while attempting to minimize the influence of swimming itself (Kelly and Bird, 2021). More recent work (Kelly, 2026; Kelly et al., 2026) further suggests that mood benefits may occur across different immersion durations and environmental contexts, although the magnitude of benefit may differ between laboratory based and natural outdoor settings. These findings raise the hypothesis that immediate mood related outcomes may be more responsive than established psychiatric symptoms. However, the present review does not establish a differential or clinically meaningful effect across these outcome domains.

The comparatively weak findings for anxiety symptoms are notable. Theoretical models suggest that CWE could function similarly to interoceptive exposure by confronting individuals with intense bodily sensations such as breathlessness, rapid heart rate, or feelings of panic, which are also central targets of exposure-based approaches for anxiety and panic symptoms (Barwood et al., 2018; Boswell et al., 2013). However, psychotherapeutic exposure interventions are usually embedded within structured cognitive framing, graded practice, and therapeutic support. In most CWE studies, the intervention was delivered primarily as a physiological stimulus rather than as a psychologically integrated treatment, which explain the limited and inconsistent anxiety effects.

Substantial heterogeneity across studies also complicates interpretation of the findings. The included studies differed in exposure modality, temperature, duration, frequency, setting, participant population, and comparator condition. Several interventions combined CWE with swimming, breathing exercises, mindfulness, or social interaction. These components may independently influence psychological outcomes for example, controlled breathing often occurs naturally during cold exposure as participants attempt to regulate the initial shock response (Eglin et al., 2015). Future studies should therefore examine how CWE interacts with related behavioral processes, rather than treating cold exposure as fully independent. This is particularly important because recent work suggests that setting and implementation influence outcomes. Kelly and colleagues (Kelly, 2026) found that both laboratory-based and sea-based CWE improved mood, but that sea-based immersion may produce larger effects. Thus, CWE may operate less like a conventional biomedical intervention than a behavioral practice embedded in challenge, mastery, novelty, nature exposure, and group belonging.

Interpretation of the evidence is further complicated by the comparator conditions used in many studies. Several controlled studies (Blades et al., 2024; Epel et al., 2025; Ketelhut et al., 2023; Slezakova et al., 2025) used active comparison conditions such as warm water immersion, exercise, mindfulness, breathing techniques, or usual care. These interventions are not inert and may themselves improve mood, reduce stress, or enhance wellbeing (Goto et al., 2018; Janssen et al., 2016; Mason et al., 2025). The smaller estimates in controlled only analyses indicate that effects specifically attributable to CWE remain uncertain. Differences between CWE and active comparison conditions may also have been reduced when the comparator included potentially beneficial behavioral components.

The risk of bias findings also warrants careful consideration. Most studies were judged to have a high, serious, or critical overall risk of bias. The absence of participant blinding was one important contributor and is inherently difficult to avoid in CWE research because participants know whether they are entering cold water. This limitation affects both randomized and nonrandomized designs and reflects wider tensions in evaluating behavioral and nonpharmacological interventions within frameworks modelled largely on pharmaceutical trials, where blinding, patent incentives, and large-scale industry funding are more feasible (Adams et al., 2026). These limitations substantially reduce confidence in the pooled estimates. The absence of formal GRADE ratings limits direct comparability with reviews using a standardized certainty framework. Confidence in the findings was instead evaluated through design specific risk of bias assessments, controlled only analyses, and qualitative consideration of inconsistency, imprecision, indirectness, and dependence on uncontrolled evidence.

The evidence primarily reflects immediate or short-term outcomes because the first assessment after the intervention was used in the primary synthesis and longer follow up was uncommon. The findings therefore provide little information about whether any changes persist after CWE is discontinued. Variation in modality, temperature, session duration, frequency, total intervention length, setting, and supervision further limits generalizability.

This review also has limitations at the review level. Although the search followed the prospectively published strategy and included four major bibliographic databases, two trial registries, and citation tracking, Scopus and the Cochrane Library were not searched. Records indexed exclusively in these sources may therefore have been missed. The evidence primarily reflects immediate or short-term outcomes because the first assessment after the intervention was used in the primary synthesis and longer follow up was uncommon. The findings therefore provide little information about whether any changes persist after CWE is discontinued.

Safety should be considered separately from psychological effectiveness. Acute cold exposure produces marked respiratory, sympathetic, and cardiovascular responses and may present greater risks for medically susceptible individuals (Castellani and Young, 2016; Knechtle et al., 2020). Because adverse events, screening procedures, and safety measures were reported inconsistently, the present review cannot establish that self-directed CWE is safe across populations or settings. Future studies should report eligibility screening, supervision, graded progression, withdrawal criteria, and adverse events systematically.

Future studies should prioritize adequately powered RCTs with active comparators, preregistration, clearer adverse event reporting, and longer follow up periods (Supplementary Material, **Supplementary Table 3**). Future updates of this review should consider formal GRADE assessment when a larger and more coherent body of comparison specific evidence is available. Systematic assessment of moderators such as immersion duration, frequency, setting, social context, and prior experience may help explain why some studies report stronger effects than others. Mechanistic outcomes related to autonomic regulation, distress tolerance, emotional regulation, self-efficacy, and behavioral activation may further clarify how CWE influences mental health. Future research should also examine whether CWE is more effective as an adjunctive behavioral practice, particularly when integrated with behavioral activation, physical activity, mindfulness, or exposure-based psychotherapy.

## 5 Conclusion

Overall, the current evidence is insufficient to determine whether CWE improves psychological outcomes in adults. Most pooled estimates were statistically inconclusive, several became smaller in controlled only analyses, and confidence in the findings is limited by substantial risk of bias and clinical heterogeneity. The evidence does not support the use of CWE as a standalone treatment for depression or anxiety disorders. Larger and more rigorous studies are needed to determine whether any clinically meaningful effects are present and under which conditions they occur.

## Data Availability

Publicly available datasets were analyzed in this study. This data can be found here: https://github.com/sschepanski/CWEMetaAnalysis.
