# Supplementary material for: Cold-water exposure and psychological outcomes in adults: a systematic review and meta-analysis
Source: Front Physiol. 2026 Sep 16;17:1926423. doi: 10.3389/fphys.2026.1926423 (PMC13624972; doi:10.3389/fphys.2026.1926423)
Supplement: Supplementary file 1 [file Supplementaryfile1.docx]

Supplementary Material

Table of Contents

[1	Search terms	2](#_Toc236074148)

[2	Table S1. Reasons for Exclusion at Full-Text Screening	3](#_Toc236074149)

[3	Table S2. Narrative-Only Outcomes (k = 1) and Excluded Domains	4](#_Toc236074150)

[4	Other Methods	11](#_Toc236074151)

[5	Risk of bias assessments	92](#_Toc236074152)

[6	Table S3. Future Research Recommendations	95](#_Toc236074153)

[7	PRISMA 2020 for Abstracts Checklist	97](#_Toc236074154)

[8	PRISMA 2020 Checklist	99](#_Toc236074155)

[9	Literature	103](#_Toc236074156)

# 1 Search terms

The search combined controlled vocabulary and free-text terms relating to CWE, cold water, and immersion. A sample Ovid MEDLINE strategy is:

1	exp Cold Temperature/

2	exp Cryotherapy/

3	1 or 2

4	exp Water/

5	3 and 4

6	((cold or arctic or ice or winter) adj3 (bath* or immersion* or shower* or swim*)).ab,kf,ti.

7	5 or 6

8	Wim Hof *. ab,kf,ti.

9	7 or 8

10	exp animals/ not humans.sh.

11	9 not 10

| 2 Table S1. Reasons for Exclusion at Full-Text Screening | | |
| --- | --- | --- |
| **Study** | **Title** | **Reason for Exclusion** |
| (Buijze et al., 2018) | An add-on training program involving breathing exercises, cold exposure, and meditation attenuates inflammation and disease activity in axial spondylarthritis | Conference abstract |
| (Kurniasari et al., 2022) | Cold Water Immersion Directly and Mediated by Alleviated Pain to Promote Quality of Life in Indonesian with Gout Arthritis: A Community-based Randomized Controlled Trial | Only single limb/partial immersion in cold water |
| (Hinkka et al., 2015) | Effects of cold mist shower on inflammatory arthritis patients; a cross-over controlled clinical trial | Conference abstract |
| (Hjorth & Rasmussen, 2024) | Cold water swimming as an add-on treatment for depression: a feasibility study | Conference abstract |
| (Hjorth et al., 2023) | Cold water swimming as an add-on treatment for depression: a feasibility study | Voluntary sauna sessions were offered prior or after every CWE session, participants could freely choose if, when, and how often |
| (Yankouskaya, Massey, et al., 2023) | The Effects of Whole-body Cold-water Immersion on Brain Connectivity Related to the Affective State in Adults Using fMRI: A Protocol of a Pre-post Experimental Design | Protocol |
| (Czarnecki et al., 2024) (this study was found based on an anonymous clinical trial registration) | Combination of Breathing Exercises, Cold Exposure, and Meditation Mitigate Psoriasis - Open Label, Randomized, Controlled Trial | Registered clinical trial |
|  |  |  |

| 3 Table S2. Narrative-Only Outcomes (k = 1) and Excluded Domains | |
| --- | --- |
| **Study** | **Narrative-Only Outcomes and Excluded Domains** |
| (Blades et al., 2024) | Daily stress rumination was measured throughout the 21-day intervention using participants’ end-of-day reflections on the most stressful event of the day. The outcome was modelled using multilevel growth analyses within the original study, which found no significant linear or quadratic change over time within either group. Participants in the active control showed no evidence of systematic increases or decreases in daily rumination. Participants in the Wim Hof Method group exhibited a trend suggesting an early decrease that later levelled off, but these within-group change trajectories were also nonsignificant. Despite this, the formal interaction terms between condition and time were statistically significant for both the linear and quadratic components, indicating that the shape of the change trajectory differed modestly between conditions. These interaction effects were very small in magnitude and did not translate to a clear or clinically meaningful divergence between groups across the intervention period. No summary statistics for daily rumination were available in the extracted dataset. As a result, the outcome could not be harmonized for effect-size estimation and remained a narrative-only finding. |
| (Buijze et al., 2016) | This large pragmatic trial compared cold showers of thirty, sixty, or ninety seconds added to usual warm showers with a warm-shower control condition. Health-related quality of life was assessed with the SF-36 physical and mental component scores at baseline and at thirty and ninety days of follow-up. Median physical and mental component scores were already high at baseline across all arms and changed only modestly in the intervention groups during follow-up, with substantial overlap with the control group. Slightly higher mental component values were observed for the intervention groups after thirty days, but these differences were small and no longer apparent at ninety days. Because only medians and interquartile ranges were reported and no parametric summaries were available, group contrasts could not be converted into effect sizes and therefore remained narrative only.  The trial also assessed sickness absence and self-reported illness days over ninety days. The original publication reported statistically significant reductions in sickness absence for all three cold shower durations compared with the control group based on negative binomial regression models. Likewise, a significant reduction in illness days was reported for the sixty-second shower condition compared with warm showers, whereas the thirty- and ninety-second durations did not differ significantly from control. Although these findings were statistically verified in the original analysis, the extracted dataset for the present review contained only sample sizes without means, standard deviations, incidence rate ratios, or confidence intervals. Because no numeric values required for meta-analytic effect-size conversion were available, neither sickness absence nor illness days could be harmonized, and both were classified as narrative-only outcomes.  None of the other secondary outcomes measured in this trial produced significant differences between intervention and control groups at either follow-up time point according to the original publication. These included multiple SF-36 subdomains beyond the composite scores, all of which lacked extractable summary statistics in the dataset used for the present review. Safety data indicated that no serious adverse events were deemed related to the cold-shower intervention, although mild cold-related sensations such as prolonged cold feeling in the body or extremities were frequent. Since adverse event reporting was qualitative and did not include numeric incidence per group in an extractable format, it could not be synthesized further.  Taken together, although Buijze et al provided robust statistical analyses within the original trial, the absence of extractable parametric outcome data in the present dataset meant that none of these endpoints could be included in the quantitative synthesis, and all remain narrative only. |
| (Ketelhut et al., 2023) | This randomized trial reported several physiological and psychological outcomes that could not be integrated into the quantitative synthesis because no other included studies assessed these constructs in a comparable manner. Heart rate variability, operationalized as root mean square of successive differences and standard deviation of normal-to-normal intervals, showed no evidence of change across the fifteen-day intervention period and did not differ between the intervention and control groups. Both indices remained broadly stable, and all effects were associated with very small effect sizes.  Pulse wave velocity, assessed as an index of vascular stiffness, also showed no measurable modification by the intervention. Resting PWV values were nearly identical before and after the intervention in both groups, and neither time nor group effects reached statistical significance. Measures of vascular and autonomic reactivity during an acute cold pressor test likewise remained unchanged. Changes in heart rate, systolic and diastolic blood pressure, and pulse wave velocity in response to the cold pressor stimulus, as well as subjective ratings of pain during the task, showed no significant time-by-group interactions and no independent time or group effects, indicating that repeated cold exposure combined with breathing and meditation exercises did not alter acute stress reactivity.  The study further examined subjective vitality, assessed as momentary feelings of energy and aliveness. Both dimensions of vitality displayed stable values over time, with no differential change between the intervention and control groups. Because these vitality scales were unique to this trial and no comparable measures were available in other included studies, they could not be pooled.  Together, the heart rate variability indices, pulse wave velocity outcomes, cold pressor reactivity parameters, and subjective vitality scales reported in Ketelhut et al. remained narrative-only endpoints in this review. |
| (Hirvonen et al., 2002) | They assessed several psychological and neurochemical outcomes that could not be incorporated into the quantitative synthesis because no comparable data were available across other studies and because the reporting format did not allow derivation of effect sizes. Alexithymia was measured with the TAS-20 at three seasonal time points in winter swimmers and controls, but only sample sizes were available in the extracted dataset, which prevented the evaluation of temporal trajectories or group differences. Psychopathology subscales from the Crown–Crisp Experiential Index were reported as combined values for swimmers and controls rather than separately, making it impossible to estimate intervention-specific effects. For this reason, both alexithymia and psychopathology were excluded from the meta-analysis.  The study also examined several neurochemical markers. Plasma noradrenaline and adrenaline showed pronounced seasonal decreases from autumn to winter in both winter swimmers and controls, with somewhat larger relative reductions in the swimmer group, but no statistically significant differences between groups were reported. Homovanillic acid representing dopamine metabolism remained stable across the season and did not differ between groups. Serotonin concentrations were consistently higher in swimmers than in controls, yet both groups exhibited a marked seasonal decline of approximately fifty percent, already evident in the winter sample. The serotonin metabolite 5-HIAA showed no significant seasonal change. Because these biochemical measures were unique to this study and insufficiently harmonized across groups, they could not contribute to the pooled quantitative analyses and are summarized narratively only. |
| (Huttunen et al., 2004) | They reported several outcomes that could not be incorporated into the quantitative synthesis because they were unique to this study and no comparable measures existed in the broader dataset. Memory was assessed using the OIRE instrument at the beginning of the winter swimming season and again four months later. Both winter swimmers and controls showed small reductions in memory scores over time, and the magnitude of this decline was similar in the two groups, resulting in nearly identical post-intervention values. Although swimmers exhibited a statistically significant within-group decrease in memory complaints, the parallel pattern in controls and the absence of harmonizable variance estimates meant that no group effect could be derived, leaving this outcome suitable only for narrative description.  The study also collected qualitative reports from participants with chronic health conditions. Several swimmers with rheumatism, asthma, fibromyalgia, or arthrosis described a subjective reduction in pain or symptom burden during the winter swimming period. These observations were anecdotal, not systematically measured, and not recorded in a way that would allow quantitative comparison with controls. They therefore remain descriptive signals rather than evaluable outcomes.  Life events were recorded as contextual information. Small numbers of participants in each group reported positive or negative life events during the study period, and the distribution of events was similar between swimmers and controls. These observations did not differ meaningfully across groups and were not reported with summary statistics that would permit effect estimation. As a result, they are included for completeness but were not eligible for quantitative synthesis. |
| (Knill-Jones et al., 2025) | They investigated cognitive and physiological responses to repeated open-water immersion in a single-group design. Several cognitive measures were collected across baseline, an acute post-immersion assessment, and four weekly follow-ups. Performance on both versions of the Stroop task remained stable throughout the protocol, with no significant changes in completion times or error rates at any time point. In contrast, performance on the Trail Making Test improved significantly. Trail Making Test A showed faster completion times in each of the four follow-up weeks compared with baseline, although no acute effect was observed immediately after the first immersion. Trail Making Test B displayed both an acute improvement after the initial immersion and continued gains across the weekly sessions. In the absence of a control group, these improvements cannot be attributed uniquely to cold-water exposure and may reflect practice effects, but they remain notable as the only cognitive measures showing systematic change.  The study also recorded physiological responses related to thermal regulation and subjective thermal experience. Skin temperature decreased markedly at every measurement point relative to baseline, indicating sustained peripheral cooling across the intervention period. Core temperature remained largely unchanged, fluctuating within a narrow range and showing no statistically significant effect of time. Thermal comfort ratings decreased consistently in all weekly assessments compared with baseline, reflecting a stable perception of cold stress during immersion sessions. Each of these temperature-related outcomes was unique to this single-group design and lacked harmonizable comparators in other studies. All these cognitive and physiological outcomes remained narrative-only endpoints because no other included trials assessed the same constructs in a way that would allow quantitative synthesis. |
| (Petraskova Touskova et al., 2022) | They reported two outcomes that could not be incorporated into the quantitative synthesis because no other included studies assessed these domains in a comparable manner. Psychopathology was measured with the TSC-40 in very small intervention and control groups. Scores in the intervention group remained low from baseline to follow-up, whereas the control group showed a higher mean score at the end of the expedition. This pattern suggested a possible divergence between groups, but the sample sizes were extremely limited and the variability within both groups was substantial. As a result, the study did not provide sufficiently robust data to quantify an intervention effect, and the TSC-40 measure remained a narrative-only outcome.  Melatonin concentrations were also measured and demonstrated significantly lower levels in the intervention group compared with the controls at the end of the expedition. Because melatonin was not assessed in any other study and the dataset contained only this single comparison, the outcome could not be harmonized for effect-size estimation. It is therefore summarized descriptively rather than included in the quantitative synthesis. |
| (Reed et al., 2023) | They conducted a single-group experiment to characterize thermal and vascular responses during and after cold-water immersion. Rectal temperature showed a clear progressive decline across the protocol, with a small but significant reduction during the early immersion phase and a further drop by thirty minutes after exiting the water. This pattern reflects a gradual cooling of core temperature despite initial stability.  A comprehensive set of vascular ultrasound measurements was also obtained. Brachial artery diameter remained unchanged during the first minute of immersion but was significantly reduced at fifteen minutes and again thirty minutes after immersion. Measures of blood velocity displayed a selective pattern: mean and antegrade velocities remained stable during immersion but were significantly reduced at thirty minutes post-immersion, whereas retrograde velocity did not change across any time point. Blood flow followed a similar trajectory, with mean and antegrade flow preserved during the initial immersion but reduced during prolonged exposure and in the post-immersion phase. Retrograde flow remained unchanged.  Indices of shear stress showed reductions only after immersion. Total shear stress and antegrade shear rate both declined significantly at thirty minutes post-immersion, while retrograde shear rate remained stable. The oscillatory shear index increased significantly in the post-immersion period, indicating a shift in flow profile, and forearm vascular conductance was markedly reduced during prolonged immersion and remained lower thirty minutes later. These vascular and thermoregulatory responses were unique to this study, lacked harmonizable comparators in other trials, and therefore remained narrative-only outcomes in the present review. |
| (Yankouskaya, Williamson, et al., 2023) | They reported several physiological and neuroimaging outcomes that were unique to this study and therefore could not be synthesized quantitatively. Respiratory responses showed marked acute changes during cold-water immersion, with breathing tidal volume increasing substantially from baseline and remaining elevated throughout the immersion period. Inspiratory responses demonstrated parallel increases, indicating a robust respiratory activation consistent with a cold-shock pattern. These measures were not collected in any other included trial and remained narrative only.  The study also provided the most detailed functional neuroimaging data among all included trials. Resting-state fMRI analyses demonstrated that cold-water immersion was associated with altered connectivity between large-scale neural networks. Significant post-immersion increases were observed in coupling between the medial prefrontal cortex of the default mode network and multiple nodes of the salience and frontoparietal networks, alongside concurrent decreases in connectivity with the anterior cingulate cortex. Additional clusters showed strengthened connectivity between the frontoparietal, dorsal attention, and visual networks. These effects were consistent across a range of connectivity thresholds, suggesting a robust and topologically coherent pattern of network reorganization following immersion.  Connectivity analyses that incorporated affective change scores further identified relationships between shifts in neural coupling and changes in positive or negative affect, although these associations could not be harmonized with outcomes from other studies. Because all neuroimaging and respiratory measures were unique to this study and lacked comparable data from other trials, they were not eligible for quantitative synthesis and are summarized narratively only. |
| (Epel et al., 2025) | In addition to the CES-D, PSS, and global mood/affect outcomes (MDES) that were included in the main quantitative synthesis, this trial reported several secondary endpoints that could not be integrated into the meta-analysis because they were analyzed exclusively via mixed-effects/growth-curve models and were not available in an extractable summary-statistics format (i.e., no group-wise means/SDs at each relevant time point, and no convertible contrast estimates such as adjusted mean differences with standard errors). Specifically, the authors assessed daily diary outcomes across the 21-day intervention period, including daily positive affect and daily negative affect (MDES-derived items), as well as daily stress rumination (end-of-day rating of perseverative thinking about the day’s most stressful event). These outcomes were modeled with multilevel growth analyses (linear and quadratic time terms, plus condition-by-time interactions). The paper reported that daily rumination did not change over time and did not differ by condition, and that daily affect showed small overall time trends with limited evidence of differential change between groups, but the necessary numeric summaries for harmonized effect-size estimation were not available in the extracted dataset; therefore, these daily endpoints remained narrative-only.  The study also assessed salivary cortisol reactivity to acute laboratory stress (TSST) pre- versus postintervention, operationalized via AUCg and AUCi derived from four salivary samples per lab visit. These cortisol outcomes were analyzed using multilevel models (including covariates such as age and BMI), and the reported results indicated no significant pre–post change in cortisol reactivity and no significant condition-by-time interactions. However, because the extracted dataset did not contain extractable group-wise parametric summaries or convertible model outputs for AUCg/AUCi, cortisol reactivity could not be harmonized for meta-analysis and remained narrative-only.  Finally, the trial measured intervention credibility, expectancy, and acceptability using the Credibility/Expectancy Questionnaire (CEQ), the Treatment Acceptability Questionnaire (TAQ), and expectation items regarding anticipated mental and physical health benefits. These process measures were used to evaluate comparability of expectancy effects across arms and were reported descriptively/through omnibus tests, but they were not part of any pooled outcome domain and were not assessed in a harmonizable way across other included studies; thus, they were classified as excluded domains (k = 1) and summarized narratively. |

# 4 Other Methods

For continuous outcomes, we calculated standardized mean differences (SMDs) using Hedges g. Depending on the available data and study design:

- **Between-group post-intervention comparisons** (RCTs, non-randomized controlled trials): SMD = (Mean_CWE - Mean_control) / pooled SD, with small-sample correction. When change scores were reported and clearly comparable, SMDs were computed from change values using the appropriate variance formulas.
- **Single-group pre-post designs**: SMD was computed as mean change divided by SD of change, with small-sample correction. When SD of change was not available, it was approximated from baseline and post SDs using an assumed pre-post correlation informed by similar studies; sensitivity checks with alternative plausible correlations were used where necessary.
- **Variance estimation**: Variances of SMDs were derived using standard formulas for independent and paired data.

When SDs were not reported but CIs, SEs, t values, or p values were available, we reconstructed SDs using methods described elsewhere (Luo et al., 2018; Wan et al., 2014). Effect sizes were oriented so that positive SMDs reflected improvements in positive outcomes (e.g., wellbeing) and negative SMDs reflected reductions in symptom measures (e.g., depression, anxiety, stress).

**Supplementary Figure S1. Depression (all designs).** Forest plot of standardized mean differences (SMD, Hedges g) for depressive symptoms across all included study designs. Negative values indicate effects favoring cold-water exposure (CWE). Squares represent study-specific effect sizes, with size proportional to inverse variance (weight), and horizontal lines denote 95% confidence intervals (CI). The diamond indicates the pooled estimate. SMD, standardized mean difference; CI, confidence interval; CWE, cold-water exposure.

**Supplementary Figure S2. Depression (controlled studies).** Forest plot of standardized mean differences (SMD, Hedges g) for depressive symptoms restricted to controlled study designs. Negative values indicate effects favoring cold-water exposure (CWE). Squares represent study-specific effect sizes (size proportional to inverse variance), with horizontal lines showing 95% confidence intervals (CI). The diamond indicates the pooled estimate. SMD, standardized mean difference; CI, confidence interval; CWE, cold-water exposure.

**Supplementary Figure S3. Depression (leave-one-out, all designs).** Leave-one-out sensitivity analysis showing pooled standardized mean differences (SMD, Hedges g) for depressive symptoms after sequential omission of each study. Negative values indicate effects favoring cold-water exposure (CWE). Points represent pooled estimates, with horizontal lines indicating 95% confidence intervals (CI). SMD, standardized mean difference; CI, confidence interval; SE, standard error; CWE, cold-water exposure.

**Supplementary Figure S4. Depression (leave-one-out, controlled studies).** Leave-one-out sensitivity analysis restricted to controlled studies, showing pooled standardized mean differences (SMD, Hedges g) for depressive symptoms after sequential omission of each study. Negative values indicate effects favoring cold-water exposure (CWE). Points represent pooled estimates, with horizontal lines indicating 95% confidence intervals (CI). SMD, standardized mean difference; CI, confidence interval; SE, standard error; CWE, cold-water exposure.

**Supplementary Figure S5. Depression (τ² sensitivity analysis).** Sensitivity analysis showing pooled standardized mean differences (SMD, Hedges g) for depressive symptoms under fixed between-study variance (τ²) assumptions. Negative values indicate effects favoring cold-water exposure (CWE). Points represent pooled estimates, with horizontal lines indicating 95% confidence intervals (CI). SMD, standardized mean difference; CI, confidence interval; SE, standard error; τ², between-study variance; CWE, cold-water exposure.

**Supplementary Figure S6. Anxiety (all designs).** Forest plot of standardized mean differences (SMD, Hedges g) for anxiety across all included study designs. Negative values indicate effects favoring cold-water exposure (CWE). Squares represent study-specific effect sizes (size proportional to inverse variance), with horizontal lines showing 95% confidence intervals (CI). The diamond indicates the pooled estimate. SMD, standardized mean difference; CI, confidence interval; CWE, cold-water exposure.

**Supplementary Figure S7. Anxiety (controlled studies).** Forest plot of standardized mean differences (SMD, Hedges g) for anxiety restricted to controlled study designs. Negative values indicate effects favoring cold-water exposure (CWE). Squares represent study-specific effect sizes (size proportional to inverse variance), with horizontal lines showing 95% confidence intervals (CI). The diamond indicates the pooled estimate. SMD, standardized mean difference; CI, confidence interval; CWE, cold-water exposure.

**Supplementary Figure S8. Anxiety (leave-one-out, all designs).** Leave-one-out sensitivity analysis showing pooled standardized mean differences (SMD, Hedges g) for anxiety after sequential omission of each study. Negative values indicate effects favoring cold-water exposure (CWE). Points represent pooled estimates, with horizontal lines indicating 95% confidence intervals (CI). SMD, standardized mean difference; CI, confidence interval; SE, standard error; CWE, cold-water exposure.

**Supplementary Figure S9. Anxiety (leave-one-out, controlled studies).** Leave-one-out sensitivity analysis restricted to controlled studies, showing pooled standardized mean differences (SMD, Hedges g) for anxiety after sequential omission of each study. Negative values indicate effects favoring cold-water exposure (CWE). Points represent pooled estimates, with horizontal lines indicating 95% confidence intervals (CI). SMD, standardized mean difference; CI, confidence interval; SE, standard error; CWE, cold-water exposure.

**Supplementary Figure S10. Anxiety (τ² sensitivity analysis).** Sensitivity analysis showing pooled standardized mean differences (SMD, Hedges g) for anxiety under fixed between-study variance (τ²) assumptions. Negative values indicate effects favoring cold-water exposure (CWE). Points represent pooled estimates, with horizontal lines indicating 95% confidence intervals (CI). SMD, standardized mean difference; CI, confidence interval; SE, standard error; τ², between-study variance; CWE, cold-water exposure.

**Supplementary Figure S11. Perceived stress (all designs).** Forest plot of standardized mean differences (SMD, Hedges g) for perceived stress across all included study designs. Negative values indicate effects favoring cold-water exposure (CWE). Squares represent study-specific effect sizes (size proportional to inverse variance), with horizontal lines showing 95% confidence intervals (CI). The diamond indicates the pooled estimate. SMD, standardized mean difference; CI, confidence interval; CWE, cold-water exposure.

**Supplementary Figure S12. Perceived stress (controlled studies).** Forest plot of standardized mean differences (SMD, Hedges g) for perceived stress restricted to controlled study designs. Negative values indicate effects favoring cold-water exposure (CWE). Squares represent study-specific effect sizes (size proportional to inverse variance), with horizontal lines showing 95% confidence intervals (CI). The diamond indicates the pooled estimate. SMD, standardized mean difference; CI, confidence interval; CWE, cold-water exposure.

**Supplementary Figure S13. Perceived stress (leave-one-out, all designs).** Leave-one-out sensitivity analysis showing pooled standardized mean differences (SMD, Hedges g) for perceived stress after sequential omission of each study. Negative values indicate effects favoring cold-water exposure (CWE). Points represent pooled estimates, with horizontal lines indicating 95% confidence intervals (CI). SMD, standardized mean difference; CI, confidence interval; SE, standard error; CWE, cold-water exposure.

**Supplementary Figure S14. Perceived stress (leave-one-out, controlled studies).** Leave-one-out sensitivity analysis restricted to controlled studies, showing pooled standardized mean differences (SMD, Hedges g) for perceived stress after sequential omission of each study. Negative values indicate effects favoring cold-water exposure (CWE). Points represent pooled estimates, with horizontal lines indicating 95% confidence intervals (CI). SMD, standardized mean difference; CI, confidence interval; SE, standard error; CWE, cold-water exposure.

**Supplementary Figure S15. Perceived stress (τ² sensitivity analysis).** Sensitivity analysis showing pooled standardized mean differences (SMD, Hedges g) for perceived stress under fixed between-study variance (τ²) assumptions. Negative values indicate effects favoring cold-water exposure (CWE). Points represent pooled estimates, with horizontal lines indicating 95% confidence intervals (CI). SMD, standardized mean difference; CI, confidence interval; SE, standard error; τ², between-study variance; CWE, cold-water exposure.

**Supplementary Figure S16. Wellbeing (all designs).** Forest plot of standardized mean differences (SMD, Hedges g) for wellbeing across all included study designs. Positive values indicate effects favoring cold-water exposure (CWE). Squares represent study-specific effect sizes (size proportional to inverse variance), with horizontal lines showing 95% confidence intervals (CI). The diamond indicates the pooled estimate. SMD, standardized mean difference; CI, confidence interval; CWE, cold-water exposure.

**Supplementary Figure S17. Wellbeing (leave-one-out, all designs).** Leave-one-out sensitivity analysis showing pooled standardized mean differences (SMD, Hedges g) for wellbeing after sequential omission of each study. Positive values indicate effects favoring cold-water exposure (CWE). Points represent pooled estimates, with horizontal lines indicating 95% confidence intervals (CI). SMD, standardized mean difference; CI, confidence interval; SE, standard error; CWE, cold-water exposure.

**Supplementary Figure S18. Wellbeing (τ² sensitivity analysis).** Sensitivity analysis showing pooled standardized mean differences (SMD, Hedges g) for wellbeing under fixed between-study variance (τ²) assumptions. Positive values indicate effects favoring cold-water exposure (CWE). Points represent pooled estimates, with horizontal lines indicating 95% confidence intervals (CI). SMD, standardized mean difference; CI, confidence interval; SE, standard error; τ², between-study variance; CWE, cold-water exposure.

**Supplementary Figure S19. Mood/Affect (all designs).** Forest plot of standardized mean differences (SMD, Hedges g) for mood/affect across all included study designs. Positive values indicate effects favoring cold-water exposure (CWE). Squares represent study-specific effect sizes (size proportional to inverse variance), with horizontal lines showing 95% confidence intervals (CI). The diamond indicates the pooled estimate. SMD, standardized mean difference; CI, confidence interval; CWE, cold-water exposure.

**Supplementary Figure S20. Mood/Affect (controlled studies).** Forest plot of standardized mean differences (SMD, Hedges g) for mood/affect restricted to controlled study designs. Positive values indicate effects favoring cold-water exposure (CWE). Squares represent study-specific effect sizes (size proportional to inverse variance), with horizontal lines showing 95% confidence intervals (CI). The diamond indicates the pooled estimate. SMD, standardized mean difference; CI, confidence interval; CWE, cold-water exposure.

**Supplementary Figure S21. Mood/Affect (leave-one-out, all designs).** Leave-one-out sensitivity analysis showing pooled standardized mean differences (SMD, Hedges g) for mood/affect after sequential omission of each study. Positive values indicate effects favoring cold-water exposure (CWE). Points represent pooled estimates, with horizontal lines indicating 95% confidence intervals (CI). SMD, standardized mean difference; CI, confidence interval; SE, standard error; CWE, cold-water exposure.

**Supplementary Figure S22. Mood/Affect (leave-one-out, controlled studies).** Leave-one-out sensitivity analysis restricted to controlled studies, showing pooled standardized mean differences (SMD, Hedges g) for mood/affect after sequential omission of each study. Positive values indicate effects favoring cold-water exposure (CWE). Points represent pooled estimates, with horizontal lines indicating 95% confidence intervals (CI). SMD, standardized mean difference; CI, confidence interval; SE, standard error; CWE, cold-water exposure.

**Supplementary Figure S23. Mood/Affect (τ² sensitivity analysis).** Sensitivity analysis showing pooled standardized mean differences (SMD, Hedges g) for mood/affect under fixed between-study variance (τ²) assumptions. Positive values indicate effects favoring cold-water exposure (CWE). Points represent pooled estimates, with horizontal lines indicating 95% confidence intervals (CI). SMD, standardized mean difference; CI, confidence interval; SE, standard error; τ², between-study variance; CWE, cold-water exposure.

**Supplementary Figure S24. Sleep (all designs).** Forest plot of standardized mean differences (SMD, Hedges g) for sleep across all included study designs. Negative values indicate effects favoring cold-water exposure (CWE). Squares represent study-specific effect sizes (size proportional to inverse variance), with horizontal lines showing 95% confidence intervals (CI). The diamond indicates the pooled estimate. SMD, standardized mean difference; CI, confidence interval; CWE, cold-water exposure.

**Supplementary Figure S25. Sleep (controlled studies).** Forest plot of standardized mean differences (SMD, Hedges g) for sleep restricted to controlled study designs. Negative values indicate effects favoring cold-water exposure (CWE). Squares represent study-specific effect sizes (size proportional to inverse variance), with horizontal lines showing 95% confidence intervals (CI). The diamond indicates the pooled estimate. SMD, standardized mean difference; CI, confidence interval; CWE, cold-water exposure.

**Supplementary Figure S26. Sleep (leave-one-out, all designs).** Leave-one-out sensitivity analysis showing pooled standardized mean differences (SMD, Hedges g) for sleep after sequential omission of each study. Negative values indicate effects favoring cold-water exposure (CWE). Points represent pooled estimates, with horizontal lines indicating 95% confidence intervals (CI). SMD, standardized mean difference; CI, confidence interval; SE, standard error; CWE, cold-water exposure.

**Supplementary Figure S27. Sleep (τ² sensitivity analysis).** Sensitivity analysis showing pooled standardized mean differences (SMD, Hedges g) for sleep under fixed between-study variance (τ²) assumptions. Negative values indicate effects favoring cold-water exposure (CWE). Points represent pooled estimates, with horizontal lines indicating 95% confidence intervals (CI). SMD, standardized mean difference; CI, confidence interval; SE, standard error; τ², between-study variance; CWE, cold-water exposure.

**Supplementary Figure S28. Cortisol (all designs).** Forest plot of standardized mean differences (SMD, Hedges g) for cortisol across all included study designs. Negative values indicate effects favoring cold-water exposure (CWE). Squares represent study-specific effect sizes (size proportional to inverse variance), with horizontal lines showing 95% confidence intervals (CI). The diamond indicates the pooled estimate. SMD, standardized mean difference; CI, confidence interval; CWE, cold-water exposure.

**Supplementary Figure S29. Cortisol (controlled studies).** Forest plot of standardized mean differences (SMD, Hedges g) for cortisol restricted to controlled study designs. Negative values indicate effects favoring cold-water exposure (CWE). Squares represent study-specific effect sizes (size proportional to inverse variance), with horizontal lines showing 95% confidence intervals (CI). The diamond indicates the pooled estimate. SMD, standardized mean difference; CI, confidence interval; CWE, cold-water exposure.

**Supplementary Figure S30. Cortisol (leave-one-out, all designs).** Leave-one-out sensitivity analysis showing pooled standardized mean differences (SMD, Hedges g) for cortisol after sequential omission of each study. Negative values indicate effects favoring cold-water exposure (CWE). Points represent pooled estimates, with horizontal lines indicating 95% confidence intervals (CI). SMD, standardized mean difference; CI, confidence interval; SE, standard error; CWE, cold-water exposure.

**Supplementary Figure S31. Cortisol (leave-one-out, controlled studies).** Leave-one-out sensitivity analysis restricted to controlled studies, showing pooled standardized mean differences (SMD, Hedges g) for cortisol after sequential omission of each study. Negative values indicate effects favoring cold-water exposure (CWE). Points represent pooled estimates, with horizontal lines indicating 95% confidence intervals (CI). SMD, standardized mean difference; CI, confidence interval; SE, standard error; CWE, cold-water exposure.

**Supplementary Figure S32. Cortisol (τ² sensitivity analysis).** Sensitivity analysis showing pooled standardized mean differences (SMD, Hedges g) for cortisol under fixed between-study variance (τ²) assumptions. Negative values indicate effects favoring cold-water exposure (CWE). Points represent pooled estimates, with horizontal lines indicating 95% confidence intervals (CI). SMD, standardized mean difference; CI, confidence interval; SE, standard error; τ², between-study variance; CWE, cold-water exposure.

**Supplementary Figure S33. β-endorphin (all designs).** Forest plot of standardized mean differences (SMD, Hedges g) for β-endorphin across all included study designs. Positive values indicate effects favoring cold-water exposure (CWE). Squares represent study-specific effect sizes (size proportional to inverse variance), with horizontal lines showing 95% confidence intervals (CI). The diamond indicates the pooled estimate. SMD, standardized mean difference; CI, confidence interval; CWE, cold-water exposure.

**Supplementary Figure S34. β-endorphin (controlled studies).** Forest plot of standardized mean differences (SMD, Hedges g) for β-endorphin restricted to controlled study designs. Positive values indicate effects favoring cold-water exposure (CWE). Squares represent study-specific effect sizes (size proportional to inverse variance), with horizontal lines showing 95% confidence intervals (CI). The diamond indicates the pooled estimate. SMD, standardized mean difference; CI, confidence interval; CWE, cold-water exposure.

**Supplementary Figure S35. β-endorphin (leave-one-out, all designs).** Leave-one-out sensitivity analysis showing pooled standardized mean differences (SMD, Hedges g) for β-endorphin after sequential omission of each study. Positive values indicate effects favoring cold-water exposure (CWE). Points represent pooled estimates, with horizontal lines indicating 95% confidence intervals (CI). SMD, standardized mean difference; CI, confidence interval; SE, standard error; CWE, cold-water exposure.

**Supplementary Figure S36. β-endorphin (τ² sensitivity analysis).** Sensitivity analysis showing pooled standardized mean differences (SMD, Hedges g) for β-endorphin under fixed between-study variance (τ²) assumptions. Positive values indicate effects favoring cold-water exposure (CWE). Points represent pooled estimates, with horizontal lines indicating 95% confidence intervals (CI). SMD, standardized mean difference; CI, confidence interval; SE, standard error; τ², between-study variance; CWE, cold-water exposure.

**Supplementary Figure S37. Pain (all designs).** Forest plot of standardized mean differences (SMD, Hedges g) for pain across all included study designs. Negative values indicate effects favoring cold-water exposure (CWE). Squares represent study-specific effect sizes (size proportional to inverse variance), with horizontal lines showing 95% confidence intervals (CI). The diamond indicates the pooled estimate. SMD, standardized mean difference; CI, confidence interval; CWE, cold-water exposure.

**Supplementary Figure S38. Pain (leave-one-out, all designs).** Leave-one-out sensitivity analysis showing pooled standardized mean differences (SMD, Hedges g) for pain after sequential omission of each study. Negative values indicate effects favoring cold-water exposure (CWE). Points represent pooled estimates, with horizontal lines indicating 95% confidence intervals (CI). SMD, standardized mean difference; CI, confidence interval; SE, standard error; CWE, cold-water exposure.

**Supplementary Figure S39. Pain (τ² sensitivity analysis).** Sensitivity analysis showing pooled standardized mean differences (SMD, Hedges g) for pain under fixed between-study variance (τ²) assumptions. Negative values indicate effects favoring cold-water exposure (CWE). Points represent pooled estimates, with horizontal lines indicating 95% confidence intervals (CI). SMD, standardized mean difference; CI, confidence interval; SE, standard error; τ², between-study variance; CWE, cold-water exposure.

**Supplementary Figure S40. Systolic blood pressure (all designs).** Forest plot of standardized mean differences (SMD, Hedges g) for systolic blood pressure across all included study designs. Positive values indicate effects favoring cold-water exposure (CWE). Squares represent study-specific effect sizes (size proportional to inverse variance), with horizontal lines showing 95% confidence intervals (CI). The diamond indicates the pooled estimate. SMD, standardized mean difference; CI, confidence interval; CWE, cold-water exposure.

**Supplementary Figure S41. Systolic blood pressure (controlled studies).** Forest plot of standardized mean differences (SMD, Hedges g) for systolic blood pressure restricted to controlled study designs. Positive values indicate effects favoring cold-water exposure (CWE). Squares represent study-specific effect sizes (size proportional to inverse variance), with horizontal lines showing 95% confidence intervals (CI). The diamond indicates the pooled estimate. SMD, standardized mean difference; CI, confidence interval; CWE, cold-water exposure.

**Supplementary Figure S42. Diastolic blood pressure (all designs).** Forest plot of standardized mean differences (SMD, Hedges g) for diastolic blood pressure across all included study designs. Positive values indicate effects favoring cold-water exposure (CWE). Squares represent study-specific effect sizes (size proportional to inverse variance), with horizontal lines showing 95% confidence intervals (CI). The diamond indicates the pooled estimate. SMD, standardized mean difference; CI, confidence interval; CWE, cold-water exposure.

**Supplementary Figure S43. Diastolic blood pressure (controlled studies).** Forest plot of standardized mean differences (SMD, Hedges g) for diastolic blood pressure restricted to controlled study designs. Negative values indicate effects favoring cold-water exposure (CWE). Squares represent study-specific effect sizes (size proportional to inverse variance), with horizontal lines showing 95% confidence intervals (CI). The diamond indicates the pooled estimate. SMD, standardized mean difference; CI, confidence interval; CWE, cold-water exposure.

**Supplementary Figure S44. Systolic blood pressure (leave-one-out, all designs).** Leave-one-out sensitivity analysis showing pooled standardized mean differences (SMD, Hedges g) for systolic blood pressure after sequential omission of each study. Positive values indicate effects favoring cold-water exposure (CWE). Points represent pooled estimates, with horizontal lines indicating 95% confidence intervals (CI). SMD, standardized mean difference; CI, confidence interval; SE, standard error; CWE, cold-water exposure.

**Supplementary Figure S45. Systolic blood pressure (leave-one-out, controlled studies).** Leave-one-out sensitivity analysis restricted to controlled studies, showing pooled standardized mean differences (SMD, Hedges g) for systolic blood pressure after sequential omission of each study. Positive values indicate effects favoring cold-water exposure (CWE). Points represent pooled estimates, with horizontal lines indicating 95% confidence intervals (CI). SMD, standardized mean difference; CI, confidence interval; SE, standard error; CWE, cold-water exposure.

**Supplementary Figure S46. Systolic blood pressure (τ² sensitivity analysis).** Sensitivity analysis showing pooled standardized mean differences (SMD, Hedges g) for systolic blood pressure under fixed between-study variance (τ²) assumptions. Positive values indicate effects favoring cold-water exposure (CWE). Points represent pooled estimates, with horizontal lines indicating 95% confidence intervals (CI). SMD, standardized mean difference; CI, confidence interval; SE, standard error; τ², between-study variance; CWE, cold-water exposure.

**Supplementary Figure S47. Diastolic blood pressure (leave-one-out, all designs).** Leave-one-out sensitivity analysis showing pooled standardized mean differences (SMD, Hedges g) for diastolic blood pressure after sequential omission of each study. Positive values indicate effects favoring cold-water exposure (CWE). Points represent pooled estimates, with horizontal lines indicating 95% confidence intervals (CI). SMD, standardized mean difference; CI, confidence interval; SE, standard error; CWE, cold-water exposure.

**Supplementary Figure S48. Diastolic blood pressure (leave-one-out, controlled studies).** Leave-one-out sensitivity analysis restricted to controlled studies, showing pooled standardized mean differences (SMD, Hedges g) for diastolic blood pressure after sequential omission of each study. Negative values indicate effects favoring cold-water exposure (CWE). Points represent pooled estimates, with horizontal lines indicating 95% confidence intervals (CI). SMD, standardized mean difference; CI, confidence interval; SE, standard error; CWE, cold-water exposure.

**Supplementary Figure S49. Diastolic blood pressure (τ² sensitivity analysis).** Sensitivity analysis showing pooled standardized mean differences (SMD, Hedges g) for diastolic blood pressure under fixed between-study variance (τ²) assumptions. Positive values indicate effects favoring cold-water exposure (CWE). Points represent pooled estimates, with horizontal lines indicating 95% confidence intervals (CI). SMD, standardized mean difference; CI, confidence interval; SE, standard error; τ², between-study variance; CWE, cold-water exposure.

**Supplementary Figure S50. Heart rate (all designs).** Forest plot of standardized mean differences (SMD, Hedges g) for heart rate across all included study designs. Negative values indicate effects favoring cold-water exposure (CWE). Squares represent study-specific effect sizes (size proportional to inverse variance), with horizontal lines showing 95% confidence intervals (CI). The diamond indicates the pooled estimate. SMD, standardized mean difference; CI, confidence interval; CWE, cold-water exposure.

**Supplementary Figure S51. Heart rate (controlled studies).** Forest plot of standardized mean differences (SMD, Hedges g) for heart rate restricted to controlled study designs. Negative values indicate effects favoring cold-water exposure (CWE). Squares represent study-specific effect sizes (size proportional to inverse variance), with horizontal lines showing 95% confidence intervals (CI). The diamond indicates the pooled estimate. SMD, standardized mean difference; CI, confidence interval; CWE, cold-water exposure.

**Supplementary Figure S52. Heart rate (leave-one-out, all designs).** Leave-one-out sensitivity analysis showing pooled standardized mean differences (SMD, Hedges g) for heart rate after sequential omission of each study. Negative values indicate effects favoring cold-water exposure (CWE). Points represent pooled estimates, with horizontal lines indicating 95% confidence intervals (CI). SMD, standardized mean difference; CI, confidence interval; SE, standard error; CWE, cold-water exposure.

**Supplementary Figure S53. Heart rate (leave-one-out, controlled studies).** Leave-one-out sensitivity analysis restricted to controlled studies, showing pooled standardized mean differences (SMD, Hedges g) for heart rate after sequential omission of each study. Negative values indicate effects favoring cold-water exposure (CWE). Points represent pooled estimates, with horizontal lines indicating 95% confidence intervals (CI). SMD, standardized mean difference; CI, confidence interval; SE, standard error; CWE, cold-water exposure.

**Supplementary Figure S54. Heart rate (τ² sensitivity analysis).** Sensitivity analysis showing pooled standardized mean differences (SMD, Hedges g) for heart rate under fixed between-study variance (τ²) assumptions. Negative values indicate effects favoring cold-water exposure (CWE). Points represent pooled estimates, with horizontal lines indicating 95% confidence intervals (CI). SMD, standardized mean difference; CI, confidence interval; SE, standard error; τ², between-study variance; CWE, cold-water exposure.

# 5 Risk of bias assessments

**Figure S55. RoB1 traffic light plot of randomized controlled trials.**

**Figure S56. ROBINS-I traffic light plot of nonrandomized interventional studies.**

**Figure S57. ROBINS-E traffic light plot of observational exposure studies.**

| 6 Table S3. Future Research Recommendations | | |
| --- | --- | --- |
| **Aspect** | **Current limitations in the literature** | **Recommendations for future studies** |
| Study design and internal validity | Many studies used nonrandomized, observational, or single group designs. Randomized trials were relatively few, often small, and frequently affected by confounding, attrition, or deviations from intended interventions. Preregistration and prospective analysis plans were uncommon. | Prioritize adequately powered, preregistered randomized trials with clearly defined primary outcomes and prespecified analyses. When nonrandomized designs are used, apply methods to reduce confounding and report protocol deviations transparently. |
| Sample size and participant selection | Most studies included small samples and highly selected participants; often healthy volunteers already interested in cold exposure. Clinical populations were underrepresented. | Conduct larger multicenter studies with realistic sample size calculations. Include more diverse and clinically relevant populations, especially groups for whom CWE is promoted as a mental health or wellbeing intervention. |
| Intervention standardization and reporting | Interventions varied substantially in modality, temperature, immersion depth, duration, frequency, total intervention length, supervision, and setting. Reporting of dose escalation, adherence, and contextual factors such as season, outdoor exposure, group participation, or accompanying physical activity was often incomplete. | Report core intervention characteristics consistently, including modality, temperature, body exposure, session duration, frequency, supervision, setting, and total intervention duration. Future studies should work toward more harmonized CWE protocols to improve comparability across studies. |
| Co-interventions and intervention specificity | Many studies combined CWE with breathing exercises, meditation, mindfulness, physical activity, or broader wellness programs, making it difficult to isolate the specific contribution of cold exposure itself. | Include comparator groups or factorial designs that can distinguish the effects of CWE from co-interventions. Researchers should clearly justify whether the goal is to test CWE alone or CWE as part of a broader mind body intervention package. |
| Comparator conditions | Comparator groups were often weak, poorly matched, or absent altogether. No treatment controls and unmatched comparison groups were common. | Use comparator conditions that account for expectancy, attention, social interaction, and nonspecific intervention effects, such as warm water exposure, thermoneutral exposure, or active wellness controls. |
| Outcome selection and harmonization | Studies often used many different instruments to assess similar constructs such as stress, mood, anxiety, or wellbeing. This limited comparability across studies and complicated evidence synthesis. Mechanistic outcomes were often exploratory and underpowered. | Prespecify a small number of validated primary outcomes for core psychological domains such as depression, anxiety, stress, wellbeing, and sleep. Future studies should work toward harmonized outcome sets and include mechanistic outcomes only when they are theory driven and adequately powered. |
| Timing of assessments and follow up | Many studies only assessed outcomes immediately after the intervention. Follow up periods were often absent or inconsistent. | Include standardized assessment points at baseline, post intervention, and follow up. Longer follow up periods are needed to determine whether effects persist and whether participants continue using CWE over time. |
| Adherence and missing data | Adherence was often poorly reported and missing data handling was inconsistent. Attrition and reasons for dropout were frequently not described in detail. | Report adherence systematically, including session completion, dose escalation, and discontinuation. Use intention to treat analyses or mixed effects models where appropriate and report missing data transparently. |
| Safety and supervision | Safety procedures, adverse event monitoring, and contraindication screening were often insufficiently described. Many interventions were unsupervised despite involving cold naïve participants or high intensity exposure. | Systematically monitor and report adverse events using predefined criteria. Screen participants for contraindications such as cardiovascular disease, arrhythmias, uncontrolled hypertension, asthma, cold urticaria, or poor cold tolerance. Studies involving high intensity exposure, clinical populations, or cold naïve participants should include supervised initiation and graded progression protocols. |
| Translational relevance | Existing studies often focused on narrow intervention formats or very specific participant groups, limiting real world applicability. | Future research should aim to identify both minimum effective CWE protocols and safe implementation strategies for different populations. This would help determine whether benefits depend on the exact format of CWE or whether a range of protocols may be similarly effective when applied consistently. |
| CWE, cold water exposure | | |

# 7 PRISMA 2020 for Abstracts Checklist

| **Section and Topic** | **Item #** | **Checklist item** | **Reported (Yes/No)** |
| --- | --- | --- | --- |
| **TITLE** | | |  |
| Title | 1 | Identify the report as a systematic review. | Yes |
| **BACKGROUND** | | |  |
| Objectives | 2 | Provide an explicit statement of the main objective(s) or question(s) the review addresses. | Yes |
| **METHODS** | | |  |
| Eligibility criteria | 3 | Specify the inclusion and exclusion criteria for the review. | Yes |
| Information sources | 4 | Specify the information sources (e.g. databases, registers) used to identify studies and the date when each was last searched. | Yes |
| Risk of bias | 5 | Specify the methods used to assess risk of bias in the included studies. | Yes |
| Synthesis of results | 6 | Specify the methods used to present and synthesise results. | Yes |
| **RESULTS** | | |  |
| Included studies | 7 | Give the total number of included studies and participants and summarise relevant characteristics of studies. | Yes |
| Synthesis of results | 8 | Present results for main outcomes, preferably indicating the number of included studies and participants for each. If meta-analysis was done, report the summary estimate and confidence/credible interval. If comparing groups, indicate the direction of the effect (i.e. which group is favoured). | Yes |
| **DISCUSSION** | | |  |
| Limitations of evidence | 9 | Provide a brief summary of the limitations of the evidence included in the review (e.g. study risk of bias, inconsistency and imprecision). | Yes |
| Interpretation | 10 | Provide a general interpretation of the results and important implications. | Yes |
| **OTHER** | | |  |
| Funding | 11 | Specify the primary source of funding for the review. | No |
| Registration | 12 | Provide the register name and registration number. | Yes |

*From:* Page MJ, McKenzie JE, Bossuyt PM, Boutron I, Hoffmann TC, Mulrow CD, et al. The PRISMA 2020 statement: an updated guideline for reporting systematic reviews. BMJ 2021;372:n71. doi: 10.1136/bmj.n71. This work is licensed under CC BY 4.0. To view a copy of this license, visit <https://creativecommons.org/licenses/by/4.0/>

# 8 PRISMA 2020 Checklist

| **Section and Topic** | **Item #** | **Checklist item** | **Location where item is reported** |
| --- | --- | --- | --- |
| **TITLE** | | |  |
| Title | 1 | Identify the report as a systematic review. | Title |
| **ABSTRACT** | | |  |
| Abstract | 2 | See the PRISMA 2020 for Abstracts checklist. | See separate PRISMA 2020 for Abstracts checklist |
| **INTRODUCTION** | | |  |
| Rationale | 3 | Describe the rationale for the review in the context of existing knowledge. | Lines 67-71 |
| Objectives | 4 | Provide an explicit statement of the objective(s) or question(s) the review addresses. | Lines 71-75 |
| **METHODS** | | |  |
| Eligibility criteria | 5 | Specify the inclusion and exclusion criteria for the review and how studies were grouped for the syntheses. | Lines 97-109 |
| Information sources | 6 | Specify all databases, registers, websites, organisations, reference lists and other sources searched or consulted to identify studies. Specify the date when each source was last searched or consulted. | Lines 83-84, 86-90 |
| Search strategy | 7 | Present the full search strategies for all databases, registers and websites, including any filters and limits used. | Supplementary Material: Search Strategy |
| Selection process | 8 | Specify the methods used to decide whether a study met the inclusion criteria of the review, including how many reviewers screened each record and each report retrieved, whether they worked independently, and if applicable, details of automation tools used in the process. | Lines 92-96 |
| Data collection process | 9 | Specify the methods used to collect data from reports, including how many reviewers collected data from each report, whether they worked independently, any processes for obtaining or confirming data from study investigators, and if applicable, details of automation tools used in the process. | Lines 121-122 |
| Data items | 10a | List and define all outcomes for which data were sought. Specify whether all results that were compatible with each outcome domain in each study were sought (e.g. for all measures, time points, analyses), and if not, the methods used to decide which results to collect. | Lines 111-120 |
|  | 10b | List and define all other variables for which data were sought (e.g. participant and intervention characteristics, funding sources). Describe any assumptions made about any missing or unclear information. | Lines 122-125 |
| Study risk of bias assessment | 11 | Specify the methods used to assess risk of bias in the included studies, including details of the tool(s) used, how many reviewers assessed each study and whether they worked independently, and if applicable, details of automation tools used in the process. | Lines 132-137 |
| Effect measures | 12 | Specify for each outcome the effect measure(s) (e.g. risk ratio, mean difference) used in the synthesis or presentation of results. | Lines 144-147 |
| Synthesis methods | 13a | Describe the processes used to decide which studies were eligible for each synthesis (e.g. tabulating the study intervention characteristics and comparing against the planned groups for each synthesis (item #5)). | Supplementary Material: Latent outcome domain classification rules |
|  | 13b | Describe any methods required to prepare the data for presentation or synthesis, such as handling of missing summary statistics, or data conversions. | Lines 147-151, Supplementary Material: Effect Size Calculation and Handling of Missing Data |
|  | 13c | Describe any methods used to tabulate or visually display results of individual studies and syntheses. | Lines 161-162 |
|  | 13d | Describe any methods used to synthesize results and provide a rationale for the choice(s). If meta-analysis was performed, describe the model(s), method(s) to identify the presence and extent of statistical heterogeneity, and software package(s) used. | Lines 152-156, 162-163, |
|  | 13e | Describe any methods used to explore possible causes of heterogeneity among study results (e.g. subgroup analysis, meta-regression). | Lines 157-158 |
|  | 13f | Describe any sensitivity analyses conducted to assess robustness of the synthesized results. | Lines 160-161 |
| Reporting bias assessment | 14 | Describe any methods used to assess risk of bias due to missing results in a synthesis (arising from reporting biases). | Lines 158-160 |
| Certainty assessment | 15 | Describe any methods used to assess certainty (or confidence) in the body of evidence for an outcome. | Lines 137-142 |
| **RESULTS** | | |  |
| Study selection | 16a | Describe the results of the search and selection process, from the number of records identified in the search to the number of studies included in the review, ideally using a flow diagram. | Lines 167-168, Figure 1 |
|  | 16b | Cite studies that might appear to meet the inclusion criteria, but which were excluded, and explain why they were excluded. | Lines 219-222, Supplementary Material, Table SX |
| Study characteristics | 17 | Cite each included study and present its characteristics. | Lines 167-170, Table 1 and 2 |
| Risk of bias in studies | 18 | Present assessments of risk of bias for each included study. | Lines 176-207, Figure 2 |
| Results of individual studies | 19 | For all outcomes, present, for each study: (a) summary statistics for each group (where appropriate) and (b) an effect estimate and its precision (e.g. confidence/credible interval), ideally using structured tables or plots. | Lines 240-270, Figure 3, Supplementary Material, Supplementary Figure 1-8 |
| Results of syntheses | 20a | For each synthesis, briefly summarise the characteristics and risk of bias among contributing studies. | Lines 176-177, 182-182, 190-200 |
|  | 20b | Present results of all statistical syntheses conducted. If meta-analysis was done, present for each the summary estimate and its precision (e.g. confidence/credible interval) and measures of statistical heterogeneity. If comparing groups, describe the direction of the effect. | Lines 240-270 |
|  | 20c | Present results of all investigations of possible causes of heterogeneity among study results. | Lines 157-158 |
|  | 20d | Present results of all sensitivity analyses conducted to assess the robustness of the synthesized results. | Lines 160-161, 241-242, 245, 247-248, 249-250 |
| Reporting biases | 21 | Present assessments of risk of bias due to missing results (arising from reporting biases) for each synthesis assessed. | Lines 158-160 |
| Certainty of evidence | 22 | Present assessments of certainty (or confidence) in the body of evidence for each outcome assessed. | Lines 137-142 |
| **DISCUSSION** | | |  |
| Discussion | 23a | Provide a general interpretation of the results in the context of other evidence. | Lines 273-274, 284-285, 285-288, 289-292, 294-296, 300-303, 329-332 |
|  | 23b | Discuss any limitations of the evidence included in the review. | Lines 356-363 |
|  | 23c | Discuss any limitations of the review processes used. | Lines 157-158 |
|  | 23d | Discuss implications of the results for practice, policy, and future research. | Lines 341-346, 348-349, 367-368, 370-374, 377-379 |
| **OTHER INFORMATION** | | |  |
| Registration and protocol | 24a | Provide registration information for the review, including register name and registration number, or state that the review was not registered. | Lines 79-81 |
|  | 24b | Indicate where the review protocol can be accessed, or state that a protocol was not prepared. | Lines 79-81 |
|  | 24c | Describe and explain any amendments to information provided at registration or in the protocol. | n/a |
| Support | 25 | Describe sources of financial or non-financial support for the review, and the role of the funders or sponsors in the review. | Line 397 |
| Competing interests | 26 | Declare any competing interests of review authors. | Line 395 |
| Availability of data, code and other materials | 27 | Report which of the following are publicly available and where they can be found: template data collection forms; data extracted from included studies; data used for all analyses; analytic code; any other materials used in the review. | Lines 406-410 |

*From:* Page MJ, McKenzie JE, Bossuyt PM, Boutron I, Hoffmann TC, Mulrow CD, et al. The PRISMA 2020 statement: an updated guideline for reporting systematic reviews. BMJ 2021;372:n71. doi: 10.1136/bmj.n71. This work is licensed under CC BY 4.0. To view a copy of this license, visit <https://creativecommons.org/licenses/by/4.0/>

# 9 Literature

Blades, R., Mendes, W. B., Don, B. P., Mayer, S. E., Dileo, R., O'Bryan, J., Fromer, E., Guan, J. Y., Cheng, S. S., Mason, A. E., Prather, A. A., & Epel, E. S. (2024). A randomized controlled clinical trial of a Wim Hof Method intervention in women with high depressive symptoms. *Compr Psychoneuroendocrinol*, *20*, 100272. <https://doi.org/10.1016/j.cpnec.2024.100272>

Buijze, G. A., Jong, H. M. Y., Kox, M., Schaardenburg, D., Vugt, R. M., Popa, C., Picckers, P., & Baeten, D. L. (2018). *SAT0284 An add-on training program involving breathing exercises, cold exposure, and meditation attenuates inflammation and disease activity in axial spondyloarthritis* (Vol. 77). <https://doi.org/10.1136/annrheumdis-2018-eular.1820>

Buijze, G. A., Sierevelt, I. N., van der Heijden, B. C., Dijkgraaf, M. G., & Frings-Dresen, M. H. (2016). The Effect of Cold Showering on Health and Work: A Randomized Controlled Trial. *PloS one*, *11*(9), e0161749. <https://doi.org/10.1371/journal.pone.0161749>

Czarnecki, J., Nowakowska-Domagala, K., & Mokros, L. (2024). Combined cold-water immersion and breathwork may be associated with improved mental health and reduction in the duration of upper respiratory tract infection - a case-control study. *Int J Circumpolar Health*, *83*(1), 2330741. <https://doi.org/10.1080/22423982.2024.2330741>

Epel, E. S., Don, B. P., Mayer, S. E., Blades, R., Mason, A. E., Fromer, E., O’Bryan, J. F., Dileo, R., Guan, J. Y., & Schilf, S. J. (2025). A randomized controlled pilot trial of low and high arousal resilience interventions for depressive symptoms. *Annals of Behavioral Medicine*, *59*(1), kaaf081. <https://doi.org/10.1093/abm/kaaf081>

Faid, T., Van Gordon, W., & Taylor, E. C. (2022). Breathing Exercises, Cold-Water Immersion, and Meditation: Mind-Body Practices Lead to Reduced Stress and Enhanced Well-Being. *Adv Mind Body Med*, *36*(3), 12-20. <https://www.ncbi.nlm.nih.gov/pubmed/36308505>

Hinkka, H., Väättänen, S., & Ala-Peijari, S. (2015). AB1195 Effects of Cold Mist Shower on Inflammatory Arthritis Patients; A Cross-Over Controlled Clinical Trial. *Annals of the Rheumatic Diseases*, *74*(Suppl 2), 1302. <https://doi.org/10.1136/annrheumdis-2015-eular.2692>

Hinkka, H., Vaattanen, S., Ala-Peijari, S., & Nummi, T. (2017). Effects of cold mist shower on patients with inflammatory arthritis: a crossover controlled clinical trial. *Scand J Rheumatol*, *46*(3), 206-209. <https://doi.org/10.1080/03009742.2016.1199733>

Hirvonen, J., Lindeman, S., Matti, J., & Huttunen, P. (2002). Plasma catecholamines, serotonin and their metabolites and beta-endorphin of winter swimmers during one winter. Possible correlations to psychological traits. *Int J Circumpolar Health*, *61*(4), 363-372. <https://doi.org/10.3402/ijch.v61i4.17494>

Hjorth, P., & Rasmussen, M. R. V. (2024). Cold water swimming as an add-on treatment for depression: a feasibility study. *European Psychiatry*, *67*(S1), S533-S534. <https://doi.org/10.1192/j.eurpsy.2024.1109> M4 - Citavi

Hjorth, P., Sikjaer, M. G., Lokke, A., Jorgensen, A. M., Jorgensen, N., Kaasgaard, D. M., & Rasmussen, M. R. V. (2023). Cold water swimming as an add-on treatment for depression: a feasibility study. *Nord J Psychiatry*, *77*(7), 706-711. <https://doi.org/10.1080/08039488.2023.2228290>

Huttunen, P., Kokko, L., & Ylijukuri, V. (2004). Winter swimming improves general well-being. *Int J Circumpolar Health*, *63*(2), 140-144. <https://doi.org/10.3402/ijch.v63i2.17700>

Huttunen, P., Rintamaki, H., & Hirvonen, J. (2001). Effect of regular winter swimming on the activity of the sympathoadrenal system before and after a single cold water immersion. *Int J Circumpolar Health*, *60*(3), 400-406. <https://www.ncbi.nlm.nih.gov/pubmed/11590880>

Ketelhut, S., Querciagrossa, D., Bisang, X., Metry, X., Borter, E., & Nigg, C. R. (2023). The effectiveness of the Wim Hof method on cardiac autonomic function, blood pressure, arterial compliance, and different psychological parameters. *Sci Rep*, *13*(1), 17517. <https://doi.org/10.1038/s41598-023-44902-0>

Knill-Jones, J., Shadwell, G., Hurst, H. T., Mawhinney, C., Sinclair, J. K., & Allan, R. (2025). Influence of acute and chronic therapeutic cooling on cognitive performance and well-being. *Physiol Behav*, *289*, 114728. <https://doi.org/10.1016/j.physbeh.2024.114728>

Kurniasari, M. D., Monsen, K. A., Weng, S. F., Yang, C. Y., & Tsai, H. T. (2022). Cold water immersion directly and mediated by alleviated pain to promote quality of life in Indonesian with gout arthritis: a community-based randomized controlled trial. *Biological research for nursing*, *24*(2), 245-258. <https://doi.org/10.1177/10998004211063547>

Luo, D., Wan, X., Liu, J., & Tong, T. (2018). Optimally estimating the sample mean from the sample size, median, mid-range, and/or mid-quartile range. *Statistical methods in medical research*, *27*(6), 1785-1805. <https://doi.org/10.1177/0962280216669183>

Petraskova Touskova, T., Bob, P., Bares, Z., Vanickova, Z., Nyvlt, D., & Raboch, J. (2022). A novel Wim Hof psychophysiological training program to reduce stress responses during an Antarctic expedition. *J Int Med Res*, *50*(4), 3000605221089883. <https://doi.org/10.1177/03000605221089883>

Reed, E. L., Chapman, C. L., Whittman, E. K., Park, T. E., Larson, E. A., Kaiser, B. W., Comrada, L. N., Wiedenfeld Needham, K., Halliwill, J. R., & Minson, C. T. (2023). Cardiovascular and mood responses to an acute bout of cold water immersion. *J Therm Biol*, *118*, 103727. <https://doi.org/10.1016/j.jtherbio.2023.103727>

Slezakova, D., Adamova, L. M., Marcek, P., Kadlic, P., Konecna, M., Valkovic, P., & Minar, M. (2025). A supplementary training program integrating cold exposure, breathing exercises and mindfulness as a complementary treatment for neuropsychological aspects of multiple sclerosis - a pilot interventional study. *Mult Scler Relat Disord*, *99*, 106450. <https://doi.org/10.1016/j.msard.2025.106450>

Wan, X., Wang, W., Liu, J., & Tong, T. (2014). Estimating the sample mean and standard deviation from the sample size, median, range and/or interquartile range. *BMC medical research methodology*, *14*(1), 135. <https://doi.org/10.1186/1471-2288-14-135>

Yankouskaya, A., Massey, H., Totman, J. J., Lai, L. H., & Williamson, R. (2023). The effects of whole-body cold-water immersion on brain connectivity related to the affective state in adults using fMRI: a protocol of a pre-post experimental design. *Bio-protocol*, *13*(17), e4794. <https://doi.org/10.21769/BioProtoc.4794>

Yankouskaya, A., Williamson, R., Stacey, C., Totman, J. J., & Massey, H. (2023). Short-Term Head-Out Whole-Body Cold-Water Immersion Facilitates Positive Affect and Increases Interaction between Large-Scale Brain Networks. *Biology (Basel)*, *12*(2), 211. <https://doi.org/10.3390/biology12020211>
